# Microbial impact on initial soil formation in arid and semiarid environments under simulated climate change

**DOI:** 10.3389/fmicb.2024.1319997

**Published:** 2024-01-17

**Authors:** Victoria Rodríguez, Alexander Bartholomäus, Kristina Witzgall, Nicolás Riveras-Muñoz, Romulo Oses, Susanne Liebner, Jens Kallmeyer, Oliver Rach, Carsten W. Mueller, Oscar Seguel, Thomas Scholten, Dirk Wagner

**Affiliations:** ^1^GFZ German Research Centre for Geosciences, Section Geomicrobiology, Potsdam, Germany; ^2^Soil Science, TUM School of Life Sciences, Technical University of Munich, Freising-Weihenstephan, Germany; ^3^Department of Geosciences, Soil Science and Geomorphology, University of Tübingen, Tübingen, Germany; ^4^Centro Regional de Investigación y Desarrollo Sustentable de Atacama (CRIDESAT), Universidad de Atacama, Copiapó, Chile; ^5^Institute of Biochemistry and Biology, University of Potsdam, Potsdam, Germany; ^6^GFZ German Research Centre for Geosciences, Section Geomorphology, Potsdam, Germany; ^7^Institute for Ecology, Chair of Soil Science, Technische Universitaet Berlin, Berlin, Germany; ^8^Department of Geosciences and Natural Resource Management, University of Copenhagen, Copenhagen, Denmark; ^9^Facultad de Ciencias Agronómicas, Universidad de Chile, Santiago, Chile; ^10^Institute of Geosciences, University of Potsdam, Potsdam, Germany

**Keywords:** arid soil, semiarid soil, manipulation experiment, climate change, initial soil formation, bacterial community

## Abstract

The microbiota is attributed to be important for initial soil formation under extreme climate conditions, but experimental evidence for its relevance is scarce. To fill this gap, we investigated the impact of *in situ* microbial communities and their interrelationship with biocrust and plants compared to abiotic controls on soil formation in initial arid and semiarid soils. Additionally, we assessed the response of bacterial communities to climate change. Topsoil and subsoil samples from arid and semiarid sites in the Chilean Coastal Cordillera were incubated for 16 weeks under diurnal temperature and moisture variations to simulate humid climate conditions as part of a climate change scenario. Our findings indicate that microorganism-plant interaction intensified aggregate formation and stabilized soil structure, facilitating initial soil formation. Interestingly, microorganisms alone or in conjunction with biocrust showed no discernible patterns compared to abiotic controls, potentially due to water-masking effects. Arid soils displayed reduced bacterial diversity and developed a new community structure dominated by *Proteobacteria*, *Actinobacteriota*, and *Planctomycetota*, while semiarid soils maintained a consistently dominant community of *Acidobacteriota* and *Proteobacteria*. This highlighted a sensitive and specialized bacterial community in arid soils, while semiarid soils exhibited a more complex and stable community. We conclude that microorganism-plant interaction has measurable impacts on initial soil formation in arid and semiarid regions on short time scales under climate change. Additionally, we propose that soil and climate legacies are decisive for the present soil microbial community structure and interactions, future soil development, and microbial responses.

## 1 Introduction

Soil formation occurs at the dynamic interface between the atmosphere, hydrosphere, and lithosphere, playing a vital role in supporting life on Earth and facilitating essential biogeochemical cycles (Amundson et al., 2007). It involves interactions among physical, chemical, and biological processes, encompassing the weathering of parent rock materials and incorporating organic matter produced by biota (Schulz et al., 2013). In the early stages of soil formation, well-adapted microorganisms colonize bare mineral substrates, establishing simple communities that undergo successional changes toward more complex species interactions closely linked to soil formation (Bajerski and Wagner, 2013; Schulz et al., 2013; Liu et al., 2019; Li et al., 2021; Yu et al., 2022). Concurrently, the presence of lichens and bryophytes, along with the initial assembly of microbial communities (bacteria, fungi, and algae), leads to the formation of biological soil crusts (BSCs). These BSCs influence the nutrient input through photosynthesis, nitrogen (N) fixation, and dust capture (Elbert et al., 2012) while stabilizing the soil structure and protecting against erosion (Seitz et al., 2017; Riveras-Muñoz et al., 2022). As soil complexity and nutrient availability increase, vascular plants establish and promote weathering processes (Calvaruso et al., 2009) and aggregate formation, which stabilizes soil structure jointly with roots in the rhizosphere and organic carbon accumulation (Erktan et al., 2016; Baumert et al., 2018). Understanding the initial role, assembly, and succession of microbial communities and their intimate associations with BSCs and vascular plants is crucial for comprehending the development of soils.

Research on the role and succession dynamics of microbial communities in arid soils with low nutrient content is limited compared to low biomass soils at volcanic eruption sites or glacier forefields, which have been more extensively studied (Bajerski and Wagner, 2013; Kelly et al., 2014; Meier et al., 2019; Hernández et al., 2020). Several studies have provided compelling evidence of the role of specific microbial species in soil development through carbon and N-fixation (Schmidt et al., 2008; Duc et al., 2009), efficient mineral weathering (Frey et al., 2010; Lapanje et al., 2012), and aggregate stabilization (Kohler et al., 2006). Furthermore, the presence of specific taxa and their predicted functions have been linked to these processes during soil development (Zhelezova et al., 2019; Krauze et al., 2021; Rodriguez et al., 2022), suggesting the significance of microbial communities in soil formation. Nevertheless, experimental evidence on the interplay between the natural microbial community and soil formation processes remains limited. For instance, investigations have shown that microbial inoculum from arable topsoil can enhance soil aggregation in artificial soils facilitated by exopolysaccharides and fungal hyphae (Pronk et al., 2012). Additionally, ubiquitous microorganisms of mediterranean soils with moisture reaching 10% of the water-holding capacity have resulted in a tenfold increase in aggregation compared to sterile conditions (Blankinship et al., 2016). However, there is a significant gap in experimental evidence regarding the impact of microbial communities in succession from initial bare soils under arid and semiarid climate conditions into soils with a plant-root system.

Such arid and semiarid environments, which cover approximately 30% of the Earth’s land surface, typically experience slow soil development due to limited soil moisture and water movement for material dissolution and leaching (Abdelfattah, 2013; Sousa et al., 2021). However, changes in precipitation patterns can have complex effects on vegetation, chemistry, mineralogy, and soil stability (Su et al., 2010; Wang et al., 2017; Sousa et al., 2021). Consequently, although soil formation is a long-term process over hundreds and thousands of years, it can be largely accelerated under optimal water availability and temperature conditions (Buchan, 2011; Szalai et al., 2021). Moreover, climate projections for the upcoming decades indicate increased extreme precipitation events in arid regions (Donat et al., 2016; Ortega et al., 2019). Therefore, there is a growing interest in investigating how modifications in temperature and precipitation influence soil formation processes in arid soils for predicting the impacts of climate change or improving land management practices (Gelybó et al., 2018).

Moisture and temperature changes notably impact soil microorganisms, particularly in arid and semiarid environments. Increased or decreased water availability affects gene copy numbers (Barnard et al., 2013), respiration and productivity (Armstrong et al., 2016), biomass (Na et al., 2019; Ouyang and Li, 2020), diversity, and community composition (Maestre et al., 2015; Stovicek et al., 2017), due to alterations in soil properties (such as soil organic carbon content) and plant biomass. Key enzymatic activities in the carbon cycle, such as cellobiohydrolase and β-glucosidase (Ouyang and Li, 2020), and in the phosphorous metabolism, such as acid phosphatase (Na et al., 2019) also respond to water availability. However, the microbial response depends on the intensity and frequency of water pulses and ambient temperature, highlighting the complexity of bacterial responses (St’ovicek et al., 2017). For instance, sudden and substantial water input in hyperarid regions can harm many soil microbial species and decrease diversity (Azua-Bustos et al., 2018), while in other studies in the desert, it did not affect diversity (Armstrong et al., 2016). As our understanding of the direction and magnitude of microbial feedback to climate change continues to advance, it becomes increasingly important to investigate their implications for soil formation.

The Chilean Coastal Cordillera offers a unique opportunity to study soil formation across a climate gradient ranging from arid to humid conditions. Specifically, the arid site has drawn significant interest as a natural laboratory due to its extreme climate conditions and unique microbial community adapted to the harsh environment (Genderjahn et al., 2018; Schulze-Makuch et al., 2018; Aguilera and Larraín, 2021; Genderjahn et al., 2021; Schulze-Makuch et al., 2021). Moreover, the arid and semiarid sites of the Chilean Coastal Cordillera share similarities in tectonic, lithologic, and pedologic factors, as well as low nutrient content and precipitation levels (Bernhard et al., 2018; Oeser et al., 2018). This makes them suitable for investigating the effects of humid conditions on initial soil formation and microorganisms.

This study tests the hypothesis that soil microbial communities, besides abiotic factors, plants, and biological soil crusts, directly speed up short-term soil formation in arid regions, especially when humidity and temperature are favorable. Additionally, we propose that soil microbial communities exhibit distinct responses to simulated climate change, with variations influenced by the soil legacy. We conduct an experimental study simulating humid conditions of the southern Coastal Cordillera and implement four treatments representing a successional gradient of soil development: sterile soil, soil with *in situ* microorganisms, soil with *in situ* microorganisms and BSCs, and soil with *in situ* microorganisms and plants. The impact of microbiota, BSCs, and plants on soil formation dynamics over time was evaluated by quantifying changes in soil physicochemical properties in the different treatments. We also characterize the diversity, structure, and interactions of the bacterial community, providing insights into their response to environmental changes.

## 2 Materials and methods

### 2.1 Study sites and sample collection

**Figure 1:**
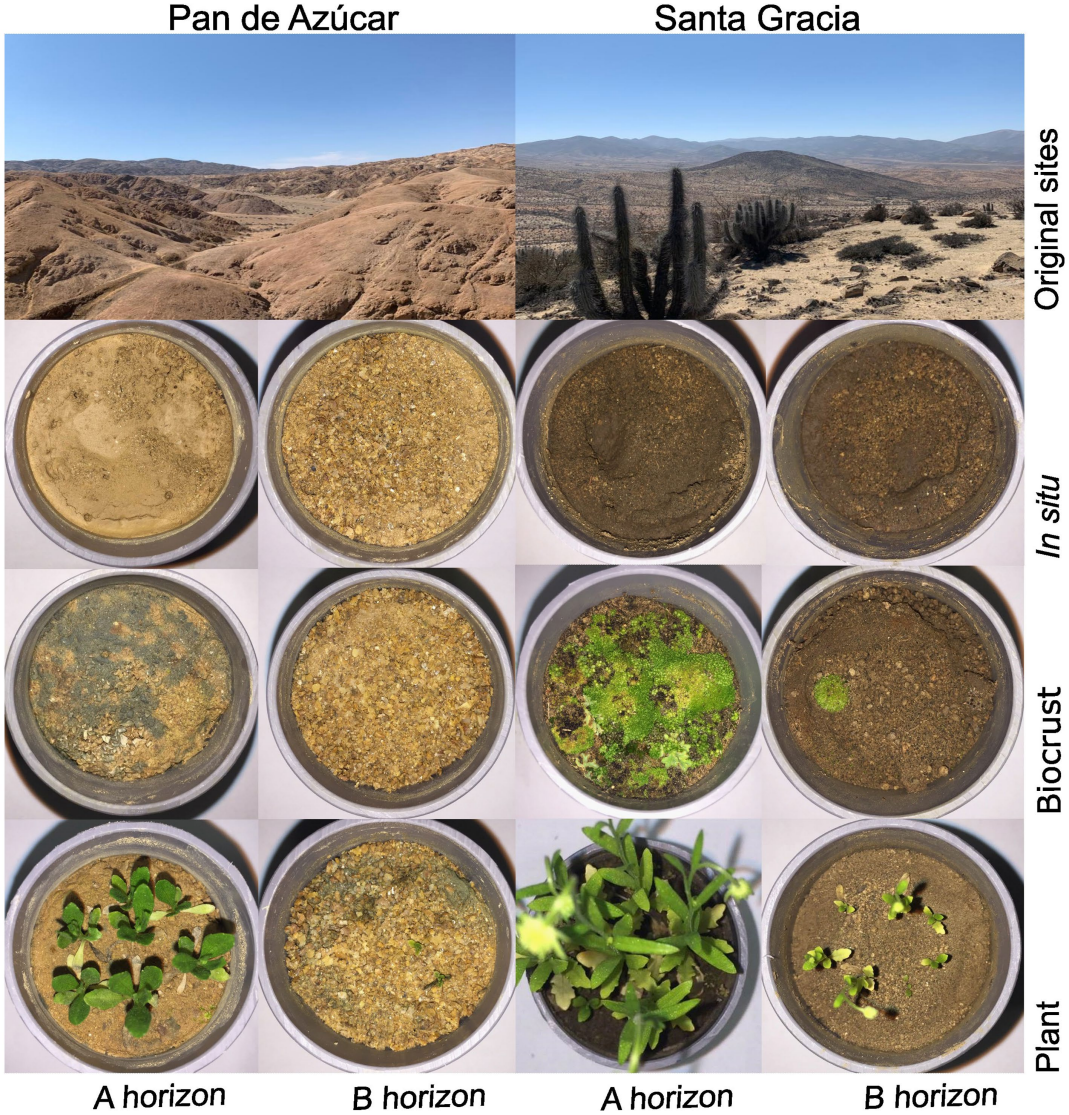
Sampling sites and microcosms of Pan de Azúcar and Santa Gracia after 12 weeks of incubation. The different microcosms are separated by horizon and treatment, including *in situ*, BSC (Biocrust), and plant treatment.

We collected soil samples from two protected areas, Pan de Azúcar National Park (PA) and Santa Gracia Natural Reserve (SG), located in the Chilean Coastal Cordillera (Figure 1, Supplementary Figure S1). The lithology at both sites is granitoid, ensuring similar parent material for soil formation and a meaningful comparison of the obtained results (Bernhard et al., 2018; Oeser et al., 2018). The mean annual precipitation and mean annual temperature are 12 mm and 16.8°C in PA and 66 mm and 13.7°C in SG, respectively (Muñoz et al., 2007). These climatic differences result in distinct soil moisture and temperature regimes. PA is arid with almost no precipitation and mostly endorheic water resources. In contrast, SG is in a semiarid zone with coastal fog influence, annual rainfall variation, and winter rain (Bernhard et al., 2018).

The field sampling campaign took place in March 2019, at the end of the austral summer season. The coordinates and elevations for PA were 26°06′35”S, 70°32′57”W, and ∼336 m elevation, while for SG, they were 29°45′15”S, 71°10′3”W, and ∼720 m elevation. Soil samples were taken from five plots devoid of vegetation and two soil horizons (A and B) for each site (Supplementary Table S1). In PA, the A horizon (PA – Ahor) ranged from 0 to 2 cm, and the B horizon (PA – Bhor) ranged from 2 to 25 cm. Similarly, in SG, the A horizon (SG – Ahor) ranged from 0 to 3 cm, and the B horizon (SG – Bhor) ranged from 3 to 40 cm. The soil structure characteristics varied between horizons. PA – Ahor exhibited a cemented crust, while PA – Bhor had a few round aggregates between coarse material, single grain dominating in the fine fraction, and gypsum concentrated in one of the plots. SG – Ahor was characterized by weak aggregates, and SG – Bhor ranged from weak to strong and stable aggregates. Fresh samples from each plot and horizon separately were homogenized through a 2 mm sieve and stored at 4°C until use.

### 2.2 Soil manipulation experiment

An experiment was performed to stimulate initial soil formation into developed soil ecosystems by applying humid climate conditions in soil microcosms, mimicking a climate change scenario. The simulated climate change was based on the climate data of Nahuelbuta National Park in the Chilean Coastal Cordillera, where we can find well-developed deep soils with a stable soil structure (Bernhard et al., 2018; Riveras-Muñoz et al., 2022). As in the natural conditions of Nahuelbuta, we applied a photoperiod of 14/10 h day/night and the daily temperature fluctuation of January (18.3/11.2°C day/night) in the soil manipulation experiment. To simulate water availability by precipitation, 25 mL of filtered and autoclaved MiliQ water was gently applied at once three days per week until 65% water-filled pore space was reached after each application. This value approximates the precipitation in Nahuelbuta (1.469 mm y^−1^) but is lower due to the water retention capacity of desert soils. The microcosms consisted of 130 g of field moist soil (sieved <2 mm and previously stored at 4°C for nine months) placed in sterile polyvinyl chloride columns (70 mm in height and 50 mm in diameter) with a silk fabric underneath to prevent soil scattering. The microcosms were arranged on a grid inside an incubator (Memmert ICP-110, Germany), mimicking the daily temperature fluctuations. The photoperiod was set using LED-SANlight S2W (IMAGRO, Germany) and controlled with a timer coordinated with temperature fluctuations.

For the manipulation experiment setup, soil material from five plots representing top and subsoil was pooled and homogenized. The experiment included 144 microcosms representing two sites (PA and SG), two soil horizons (Ahor and Bhor), four treatments, three sampling times, and three biological replicates for each combination (Supplementary Figure S2). In addition, three original, untreated samples were collected as baseline measurements for each site and horizon (T0), totaling 156 samples. The treatments consisted of a successional gradient of soil development as follows: (i) *in situ* samples with indigenous microorganisms but without BSCs or plants, (ii) *in situ* samples with naturally growing BSCs to assess the impact of microorganisms at a later stage of soil development, (iii) *in situ* samples with *Helenium aromaticum* (Hook.) L.H. Bailey (pioneer plant, described below) to simulate later stages of soil development and (iv) abiotic controls based on soil sterilized samples with ^60^Co as a source of gamma rays and a dose of 50 kGy (BGS Beta–Gamma–Service GmbH and Co. KG, Wiehl, Germany) to exclude the impact of indigenous microorganisms. *H. aromaticum* is a native vascular annual herb widely distributed in Chile, known as a pioneer plant that colonizes disturbed soils, forming seasonal grasslands (Gomez-Gonzalez et al., 2011). For plant preparation, the seeds were sterilized using 20% v/v sodium hypochlorite and 0.05% Tween 20, washed three times with autoclaved MiliQ water, and placed in sterile petri dishes with absorbent paper for germination. The germinated seeds were subsequently transferred to the microcosms. BSCs occurred naturally in the soil, whereas *in situ* samples with microorganisms were covered with a black breathable cloth to inhibit their growth.

Microcosms were sampled at 2 weeks (T1), 12 weeks (T2), and 16 weeks (T3) of incubation. Forty eight samples were sacrificed for each sampling time, covering two sites, two soil horizons, four treatments, and three replicates. In the 12th week of sampling, the plants were harvested, and the soil with the roots was retained until the 16th week of the experiment to assess their effects on the soil. Three microcosms were transferred to a laminar flow chamber for each treatment and time point to ensure sterile conditions during sub-sampling. From the total sample, 80 g of soil was collected for soil physicochemical analysis and stored in the dark at room temperature. Additionally, 10 g of soil was taken for DNA extraction and held at −20°C, while 40 g was stored at-80°C for future RNA extraction and subsequent analysis.

### 2.3 Soil physicochemical properties

To determine initial soil formation, the aggregate size distribution, and the content of total carbon (C_t_) and total nitrogen (N_t_) by aggregate size, we collected a total of 104 soil samples from three different sampling points (T0, T1, and T3). For each sample, 5 g of dry soil was shaken using a modified Casagrande apparatus (Mennerich Geotechnik, Hannover, Germany). The apparatus consisted of two sieves with mesh sizes of 250 μm and 53 μm, placed on top of a collecting vessel. The soil samples were subjected to 1,000 repeated cycles of sieving at a frequency of 2 Hz, resulting in the separation of three aggregate size classes: macroaggregates (> 250 μm), large microaggregates (250–53 μm), and small microaggregates and primary particles (< 53 μm). The data on aggregate size distribution was used to estimate aggregate stability given by the mean weight diameter of the aggregates (ΔMWD) as follows:


$$\Delta MWD=\sum_{i=1}^{n}X_{i}*W_{i}$$

*W_i_* is the corrected mass proportion of stable aggregate fraction *i* in the total aggregates (< 2 mm) and *X_i_* is the mean diameter of stable aggregate fraction *i*.

Subsequently, ball milling was conducted before measuring the content of C_t_ and N_t_ in all aggregate size classes. The process involved milling the samples for 3 min at an oscillation frequency of 30 Hz. C_t_ and N_t_ measurements for all fraction samples were performed using a CHNSO elemental analyzer (HEKAtech Euro EA, Wegberg, Germany).

Additionally, we determined the total organic carbon (TOC) and total nitrogen in the bulk soil (N_bulk_) without aggregate size separation. This analysis used 6 mg of bulk soil from the same subset of soil samples (104). The measurements were carried out using a flash HT 2000 Organic elemental analyzer, following a 6 M chlorhydric acid digestion of carbonate at 70°C.

Soil pH and electrical conductivity (EC) were determined for all samples using 15 g of soil. The soil pH was measured using a WTW pH 340 (WTW GmbH, Weilheim, Germany) with a Sentix 81 electrode. EC was measured using a conductivity meter (LE703, Mettler Toledo, United States). For inorganic ion analyses, 5 g of each soil sample was dried for 12 h at 50°C and ground to a finer consistency. Then, the leaching method described by Genderjahn et al. (2018) was employed to extract the ions from the soil. Subsequently, the resulting leachate was measured using ion chromatography (IC). The anions investigated included chloride, nitrate, phosphate, and sulfate, while the cations analyzed were sodium and calcium. Ion concentrations were calculated based on standards (Roth, Multi-Element IC-Standard Solution) using ChromStar7 software. All analyses were performed in triplicate.

### 2.4 DNA extraction and quantitative PCR analysis

Total genomic DNA was extracted in duplicates from 0.5 g of soil for PA and 0.25 g for SG using the Power Soil DNA extraction kit (Qiagen, Hilden, Germany), following the protocol described by the manufacturer. DNA extraction was exclusively performed on samples subjected to treatments involving microorganisms (*in situ*, biocrust, and plant treatments), resulting in a total of 116 samples. Sterile treatments devoid of biological relevance were excluded from the DNA extraction. The abundance of bacterial subunit 16S rRNA gene copies was determined by quantitative PCR (qPCR), following the procedure of Bernhard et al. (2018). qPCR assays were performed on a CFX 96 Connect Real-Time System (Bio-rad Laboratories, CA, United States) in 96-well plates. The primers Eub341F (5′-ATT ACC GCG GCT GCT GG-3′) and Eub534R (5′-CCT ACG GGA GGC AGC AG-3′) (Muyzer et al., 1993) were used. The qPCR mixture consisted of 10 μL KAPA SYBR Fast qPCR Kit Master Mix (2x) Universal (Kapa Biosystems, Sigma-Aldrich, Germany), 0.4 μL of each forward and reverse primer, 4.2 μL of DNA-free water, and 5 μL of extracted soil DNA template, resulting in a total volume of 20 μL. The PCR conditions were as follows: initial denaturation at 95°C for 3 min, followed by 40 cycles of denaturation at 95°C for 3 s, annealing at 60°C for 20 s, and elongation at 72°C for 30 s. We performed a melting curve analysis from 65°C to 95°C, with an increment of 0.5°C to ensure the qPCR specificity. We used standard curves from a known number of PCR-amplified target genes of a pure culture of *Escherichia coli* to estimate the unknown bacterial abundance. All the samples were analyzed in three replicate reactions.

### 2.5 Sequencing

For sequencing, DNA extracted from each sample was amplified using the primer pair 515F (GTG YCA GCM GCC GCG GTA A) and 806R (GGA CTA CNV GGG TWT CTA AT), targeting the V4 hypervariable regions of the bacterial 16S rRNA gene (Apprill et al., 2015; Parada et al., 2016) including additional barcodes. The polymerase chain reactions were performed in triplicate in a total volume of 25 μL, following the conditions described in Rodriguez et al. (2022) with modifications (reducing the number of denaturation cycles to 20 instead of 30). To confirm the amplification success, PCR products were analyzed by electrophoresis in 1.5% agarose gels stained with GelredTM Nucleic acid gel stain (Biotium, United States). The triplicate PCR samples were pooled and then purified using the carboxyl-coated magnetic beads (Agencourt^®^ AMPure^®^ XP Kit, Beckman Coulter, Brea, CA, United States). The purified DNA was quantified using the Qubit Fluorometer (InvitrogenTM, Thermo Fisher Scientific, United States). For sequencing, all PCR products were pooled in equal concentrations of 20 ng for Paired-end sequencing (2× 300 bp) at Eurofins (Eurofins Genomics Europe Shared Services GmbH, Ebersberg, Germany).

### 2.6 Processing of 16S rRNA data

To generate the amplicon sequence variants (ASVs), the sequencing library was first demultiplexed using cutadapt v3.4 (Martin, 2011) with the following parameters: -e 0.2-q 15,15-m 150 –discard-untrimmed. Only read pairs with the correct barcodes at both ends were retained for further analysis. Next, the DADA2 package v1.20 (Callahan et al., 2016) in R v4.1 was employed to generate ASVs from the trimmed reads using a pooled approach with the following parameters: truncLen = c(240,200), maxN = 0, rm.phix = TRUE, minLen = 200. Taxonomic assignment was performed using DADA2 and the SILVA database v138 (Quast et al., 2012). To filter out non-target sequences, ASVs representing chloroplasts, mitochondria, and singletons were removed from the dataset. The raw Illumina sequencing data have been deposited at the European Nucleotide Archive under the BioProject ID PRJEB60029.

### 2.7 Data analysis

All statistical analyses were conducted in R Studio v4.0.5. We performed the Kruskal-Wallis test to determine differences in soil properties, the relative abundance of 16S rRNA, and diversity indices. Post-hoc pairwise comparisons were performed using the Mann–Whitney test, with *p-*values adjusted to Bonferroni correction for multiple comparisons.

To minimize bias due to sequencing depth, all samples were rarefied to 12,000 reads per sample using the rtk package (Saary et al., 2017). Alpha diversity indices, including the Shannon index, observed species, and evenness, were calculated using the vegan package (Oksanen et al., 2013). Relative abundances were calculated by transforming absolute read numbers, and soil physicochemical properties were normalized by subtracting the mean and dividing by the standard deviation. Nonmetric multidimensional scaling (NMDS) with Bray-Curtis distance dissimilarity was performed to assess differences in bacterial structure. The significance of differences between sites, horizon, and time was estimated using PERMANOVA (adonis function) and post-hoc pairwise comparisons (pairwise.adonis) from the pairwiseAdonis package (Martinez Arbizu, 2017) with a significance of *p* < 0.05. Soil properties influencing bacterial composition were determined using distance-based redundancy analysis (dbRDA) with the capscale function in vegan. The multicollinearity of these variables was examined using the variance inflation factor (VIF), and values >10 were removed. Finally, the FAPROTAX database (Louca et al., 2016) was used to analyze the whole bacterial community and predict function from taxonomy. Correlation analyses between molecular analysis and soil properties were performed using Pearson correlation (*p <* 0.05). The results were visualized using the ggplot2 package (Wickham, 2011).

For further analyses, only abundant ASVs with a proportion of total sequences greater than 0.05% were retained to reduce underrepresented ASVs and table complexity. Indicator species were identified using the indval function in the labdsv package to find ASVs leading to changes in multivariate patterns (Roberts and Roberts, 2016). Associations were considered significant with an *R*-value of 0.8 (*p* < 0.05). Then, a network analysis was conducted to understand the distribution patterns of bacterial assemblages using a simplified version of the ecological interactions (Barberan et al., 2012; Qian et al., 2018). The co-occurrence network was constructed using the igraph package (Csardi and Nepusz, 2006) and visualized through the Fruchterman-Reingold layout. Pairwise correlations among ASVs were calculated using the rcorr function within the Hmisc package (Harrell and Harrell, 2019). A Pearson correlation coefficient of *R*-value >0.7 (*p* < 0.01) between two ASVs was considered statistically robust. Topological properties of the network, such as the number of nodes and edges, clustering coefficient, average degree, connectance, and modularity, were calculated using the igraph package. Furthermore, clusters of highly correlated ASVs were grouped into modules using the WGCNA package (Langfelder and Horvath, 2008), and their associations with physicochemical properties were explored. Highly connected nodes (hub ASVs) were then identified from each module.

## 3 Results

### 3.1 Soil manipulation experiment

The microcosms represent a successional gradient of soil development divided into three biological treatments (Figure 1, detailed in Supplementary Figure S3): soil with *in situ* microorganisms (*in situ*), soil with *in situ* microorganisms and BSCs (BSC), and soil with *in situ* microorganisms and plants (plant). The *in situ* treatment had no plant growth or BSC development, as well as the sterile treatment. In the BSC treatment, varying types and coverage were observed among sites and horizons. Specifically, a leaden BSC developed in PA, while a greenish BSC formed in SG in both horizons. The A horizons of both sites showed nearly complete coverage of the microcosms with BSCs. In contrast, the B horizons showed patchy coverage without forming a compact or extended layer of BSC on the surface. Regarding the plant treatments, similar patterns were observed. In PA, the roots were light brown, and the biomass (both below-and aboveground) and vegetative development were comparatively lower than in SG. In contrast, SG showed dark brown roots, higher biomass, and reached the flowering stage in both horizons. In the PA- Ahor, plants with numerous lateral roots ranged from 2 to 3 cm in height. Conversely, in PA – Bhor, plants were less than 5 mm tall, had a short taproot with few lateral roots, and experienced a mortality rate of over 50% after seven weeks. In SG – Ahor, flowering plants with heights ranging from 11 to 14 cm were observed. These plants had a 7 cm long taproot and numerous lateral roots covering completely the microcosms. On the other hand, SG – Bhor exhibited plants ranging from 1 to 3 cm in height, with flower buds and roots reaching 5 cm in length with fewer lateral roots than SG – Ahor. After removing the plants at 12 weeks, no visual differences were observed in the roots between weeks 12 and 16, which remained compact and defined with no signs of degradation. Given the intact nature of the roots during this period, it is considered that these conditions did not impact the short-term degradation of this organic material. Overall, the differences between treatments were less pronounced in the B horizons than in the A horizons, indicating more favorable conditions for BSC and plant development in the A horizons.

### 3.2 Soil physical and chemical properties

Soil samples were analyzed to determine the mass distribution of macro-and microaggregates and the concentrations of C_t_ and N_t_ in each soil aggregate size (Supplementary Table S2). No significant differences in aggregate size distribution were found between the study sites (PA – SG; n = 54), with average proportions of 67% macroaggregates, 27% large microaggregates, and 7% small microaggregates. However, significant differences existed between horizons within each site (Ahor-Bhor; n = 27, *p* < 0.01). In both sites, the proportion of macroaggregates was highest in the B horizon, while microaggregates dominated the A horizon. Specifically, macroaggregates mean values were 54% in PA – Ahor, 81% in PA – Bhor, 63% in SG – Ahor, and 70% in SG – Bhor. For microaggregates, the means were 23% in PA – Ahor, 9% in PA – Bhor, 19% in SG – Ahor, and 15% in SG – Bhor (The full dataset is available in Supplementary Table S2). Significant differences in the concentration of C_t_ and N_t_ were observed between sites and horizons (*p* < 0.01). C_t_ was higher in macro-and large microaggregates of PA (mean 1.2%) than in SG (mean 0.7%), while N_t_ was significantly higher in macro-and microaggregates of SG (mean 0.08%) than in PA (mean 0.03%). Analysis between horizons showed that macro-and microaggregates of PA – Bhor had a higher C_t_ mean of 2% than PA – Ahor’s 0.8%. Conversely, SG – Ahor’s macro-and large microaggregates had a higher C_t_ mean of 1.1% than SG – Bhor’s 0.8%. In terms of N_t_, higher percentages were observed in SG – Ahor (mean 0.1%) and PA – Ahor (mean 0.033%) than in SG – Bhor (mean 0.07%) and PA – Bhor (mean 0.026%).

**Figure 2:**
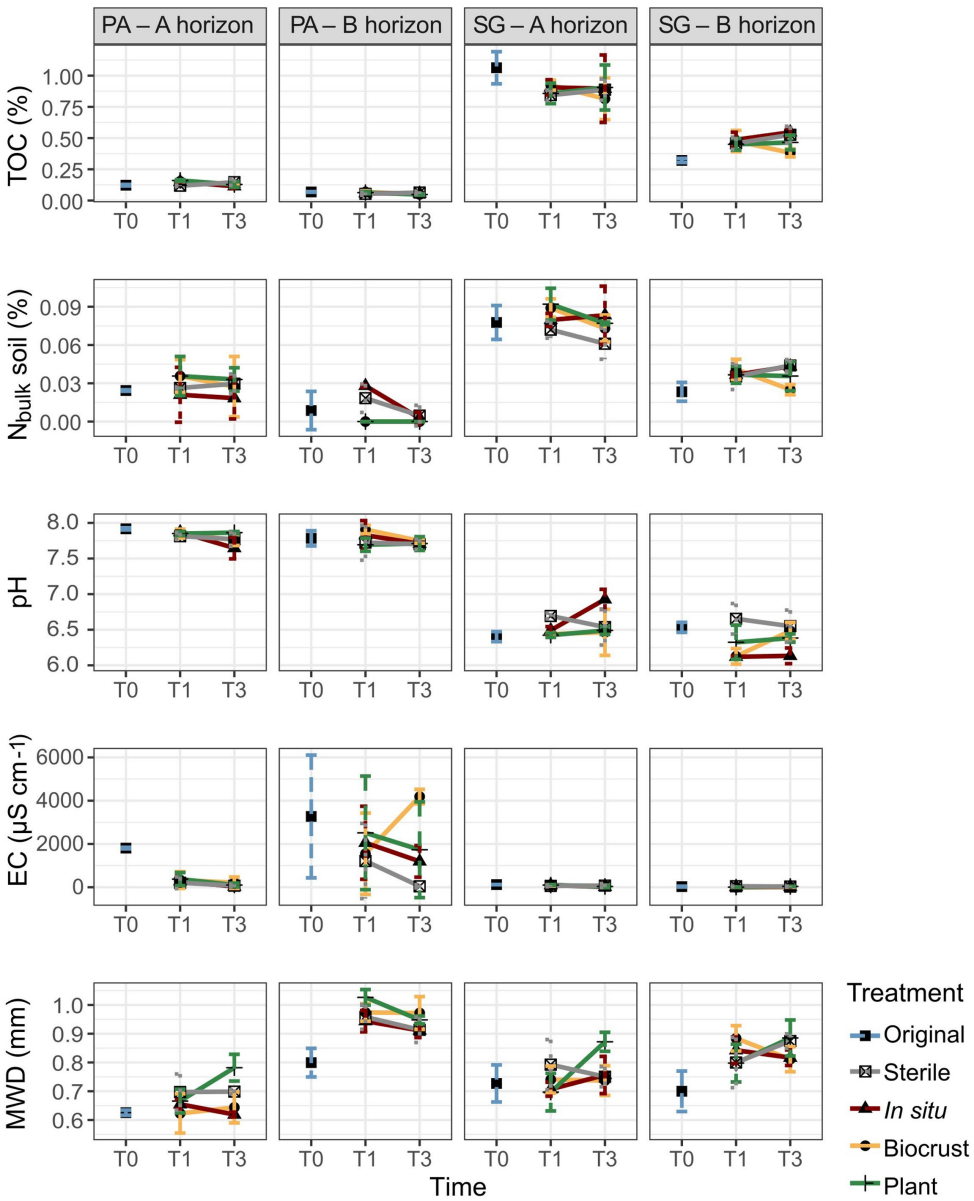
Physicochemical properties in Pan de Azúcar (PA) and Santa Gracia (SG) show total organic carbon (TOC), nitrogen in bulk soil (N_bulk_), pH, electrical conductivity (EC), and mean weight diameter (MWD) in three-time points of the soil manipulation experiment. Time is represented by T0 (original soil sample), T1 (2 weeks), and T3 (16 weeks). Different colors represent the different treatments. Each bar is the mean of biological triplicates.

Over time, we observed changes in aggregate size distribution as well as in C_t_ and N_t_ concentrations (Supplementary Table S2). In PA – Ahor, the C_t_ of large microaggregates decreased from 0.93% in T0 to 0.83% in T3. In PA – Bhor, the proportion of macroaggregates increased from 67 to 81%, while for large microaggregates decreased from 28 to 14% between T0 and T3. Consequently, MWD increased from 0.8 mm in T0 to 0.94 mm in T3. In SG – Ahor, no marked contrast was observed from T0 to T3, while in SG – Bhor, the percentage of C_t_ in macroaggregates increased from 0.2 to 0.3% over time. Regarding differences between treatments, microorganism-plant treatments showed a noticeable increase in macroaggregates and, thus, in MWD compared to the sterile treatments at T3 (Figure 2). The MWD values for sterile compared to plant treatments were as follows: PA – Ahor (mean 0.7 vs. 0.78 mm), PA – Bhor (mean 0.91 vs. 0.95 mm), SG – Ahor (mean 0.75 vs. 0.87 mm), and SG – Bhor (mean 0.87 vs. 0.89 mm).

Regarding TOC and N_bulk_ (without aggregate size separation) in the bulk soil samples, SG exhibited significantly higher levels (0.7 and 0.06%, respectively) than PA (0.1 and 0.02%, respectively). When considering the horizons, both N_bulk_ and TOC were significantly higher in PA – Ahor (0.03 and 0.14%, respectively) and SG – Ahor (0.08 and 0.9%, respectively) than PA – Bhor (0.01 and 0.06%, respectively) and SG – Bhor (0.04 and 0.46%, respectively). Over time, a notable increase in TOC was observed only in SG – Bhor, rising from 0.32% in T0 to 0.48% in T3. No clear trends were identified for the treatments (Figure 2, Supplementary Table S2).

The results revealed significant differences in soil pH, EC, and ion concentrations among the studied sites and horizons (*p* < 0.01; Figure 2, Supplementary Table S2). Specifically, in PA, higher values were observed for pH (mean 7.8), EC (mean 1,272 μS cm^−1^), sodium (mean 93.4 mg L^−1^), chloride (mean 160.2 mg L^−1^), and sulfate (mean 783.6 mg L^−1^) compared to SG, where the respective mean values were 6.5, 51.8 μS cm-1, 5.5 mg L^−1^, 6.2 mg L^−1^, and 1.4 mg L^−1^. Furthermore, significant differences in soil parameters were found between soil horizons. PA – Bhor exhibited higher EC (mean 2,186 μS cm^−1^), sodium (mean 141 mg L^−1^), chloride (mean 246.7 mg L^−1^), calcium (mean 630 mg L^−1^), and sulfate (mean 1,532.6 mg L^−1^) concentrations than PA – Ahor, where the respective mean values were 359 μS cm^−1^, 45.8 mg L^−1^, 73.8 mg L^−1^, 12.5 mg L^−1^, and 14.8 mg L^−1^. In SG, higher pH and EC were observed in the A horizon (mean 6.6 and 63 μS cm^−1^, respectively) than in the B horizon (mean 6.4 and 41 μS cm^−1^, respectively). Additionally, changes over time were observed, with substantial decreases of EC (from 1,809 to 103,7 μS cm^−1^), sodium (from 108.8 to 32.7 mg L^−1^), and chloride (from 265.3 to 48.2 mg L^−1^) concentrations from T0 to T3 in PA – Ahor. PA – Bhor also showed a marked decrease in sodium and chloride concentration from 250.2 and 522.6 mg L^−1^ to 109 and 164.5 mg L^−1^, respectively. No remarkable changes were found for the horizons of SG over time, as well as for treatments in PA or SG.

### 3.3 Bacterial abundance

**Figure 3:**
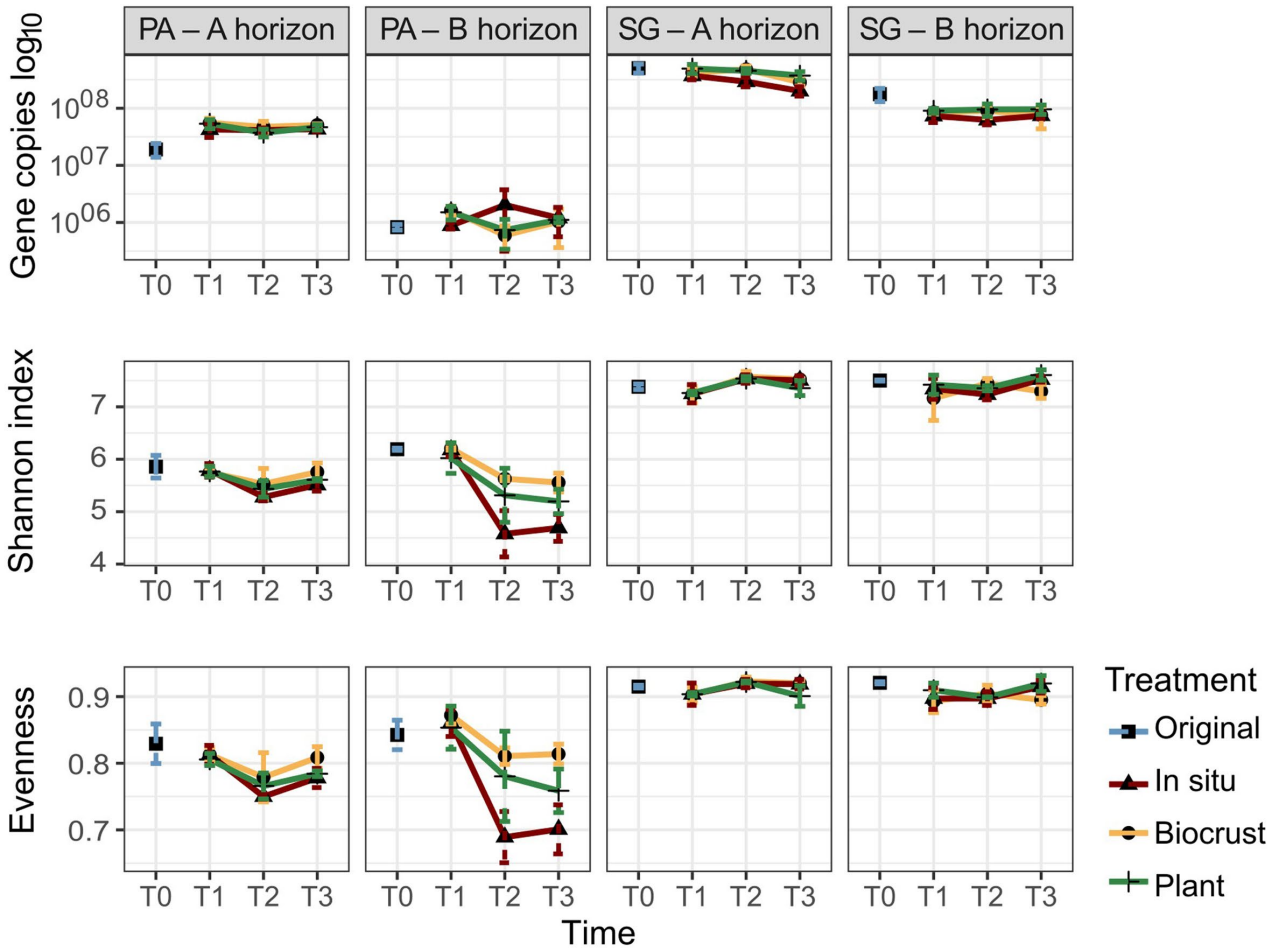
Gene copy numbers, Shannon index, and Evenness of bacterial communities in Pan de Azúcar (PA) and Santa Gracia (SG) in four soil manipulation experiment time points. Time is represented by T0 (original), T1 (2 weeks), T2 (12 weeks), and T3 (16 weeks). Different colors represent the different treatments. Each bar is the mean of biological triplicates.

The bacterial gene copy numbers are shown in Figure 3 and Supplementary Table S3. A significantly higher gene copy number for bacteria was observed in SG, ranging from 10^7^ to 10^8^ gene copies g^−1^ soil, compared to PA, ranging from 10^5^ to 10^7^ gene copies g^−1^ soil. Comparison between soil horizons showed significantly higher gene copy numbers in the A horizon for both sites. The mean of gene copies g^−1^ soil for PA – Ahor was 4.5 × 10^7^ and for PA – Bhor 1.2 × 10^6^, while for SG – Ahor was 3.9 × 10^8^ and for SG – Bhor 9.2 × 10^7^. Considering the times evaluated, both horizons in PA showed an increase in the gene copy numbers from T0 (mean of gene copies g^−1^ soil for Ahor 1.9 × 10^7^ and Bhor 8.3 × 10^5^) to T3 (mean of gene copies g^−1^ soil for Ahor 4.7 × 10^7^ and Bhor 1.1 × 10^6^). In contrast, SG decreased the gene copy number over time in both horizons. The mean of gene copy g^−1^ soil for SG – Ahor decreased from 5 × 10^8^ in T0 to 2.9 × 10^8^ in T3, while SG – Bhor reduced from 1.8 × 10^8^ in T0 to 8.2 × 10^7^ in T3. There were no discernible patterns between treatments. The gene copy numbers correlated positively with N_t_ in all aggregate classes, N_bulk_ and TOC (*R* > 0.7), and negatively with pH (*R* = 0.6).

### 3.4 Alpha and beta diversity

Sequencing analysis of 116 soil samples yielded 4.3 million raw reads, of which 3.1 million (72.7% of the total) were retained after filtering, merging, and removing chimera (Supplementary Table S4). These reads were assigned to bacteria (98.5%), archaea (1.3%), or unclassified (0.2%) groups. Since the percentage of archaea was low (with a mean of 594 reads per sample), only bacterial data were analyzed. The number of reads per bacterial sample ranged from 12,178 to 275,292, with a mean of 90,155 reads per sample. Considering the minimum read number, the samples were rarified to 12,000 reads. The final dataset contained 24,465 amplicon sequence variants (ASVs) and 1,108 putative genera.

This study evaluated the alpha-diversity indices, including the Shannon index, observed species, and evenness (Figure 3, Supplementary Table S3). The results revealed significant differences between PA and SG, with SG exhibiting higher values for all three indices. The Shannon index ranged from 4.1 to 6.3 in PA, while SG showed values ranging from 6.7 to 7.7. A similar trend was noticed for the observed species, with PA ranging from 550 to 1,754 and SG ranging from 2,075 to 4,029. For evenness, values varied between 0.87 to 0.93 in SG and 0.64 to 0.88 in PA. For the soil horizons, only the observed species values showed significant differences. PA – Ahor had a higher mean of 1,215 compared to 1,050 in PA – Bhor, whereas SG – Bhor showed a mean of 3,541 compared to 3,389 in SG – Ahor. Over time, greater changes were observed in both horizons of PA. Samples analyzed at T0 in PA – Ahor showed a marked decrease in the Shannon index and evenness at T2. The mean Shannon index decreased from 5.9 to 5.4, while evenness decreased from a mean of 0.83 to 0.76 in the same period. In PA – Bhor, the Shannon index, evenness, and observed species prominently reduced from T0 to T3. The mean Shannon index decreased from 6.2 to 5.1, evenness from 0.84 to 0.76, and the observed species mean varied from 1,568 in T0 to 892 in T3. No robust differences were observed between time or treatments in SG. However, in PA – Bhor, the Shannon index showed considerable disparities between treatments at T3, with the lowest values observed in the *in situ* treatment (4.7), followed by plants (5.2) and BSC (5.6).

Correlations between alpha diversity indices and soil properties were examined, revealing a positive correlation between the Shannon index and the observed species with N_t_ across all aggregate sizes, N_bulk_, and TOC (*R* > 0.6). Conversely, a negative correlation was observed with sodium, sulfate (*R* < −0.6), and pH (*R* < −0.9).

Analysis of NMDS showed the patterns of change in the bacterial community, and PERMANOVA allowed the calculation of the statistical differences associated with these changes (Supplementary Table S5). The statistical analysis revealed that bacterial composition was significantly different between the sites (PA vs. SG) and between the horizons (Ahor vs. Bhor; *p* < 0.01). In addition, it became visible that climate manipulation had changed the bacterial community over time (n = 9, *p* < 0.05). For both PA – Ahor and PA – Bhor, T1 was significantly different from T2 and T3. In SG – Ahor, T1 differed significantly from T3, while SG – Bhor showed no change over time. Statistical analysis with T0 was not possible due to the small number of samples (n = 2). PA – Ahor, PA – Bhor, and SG – Ahor treatments showed increasing disparity, transitioning from initial similarity to marked dissimilarity among the treatments as time progressed (Supplementary Figure S4).

**Figure 4:**
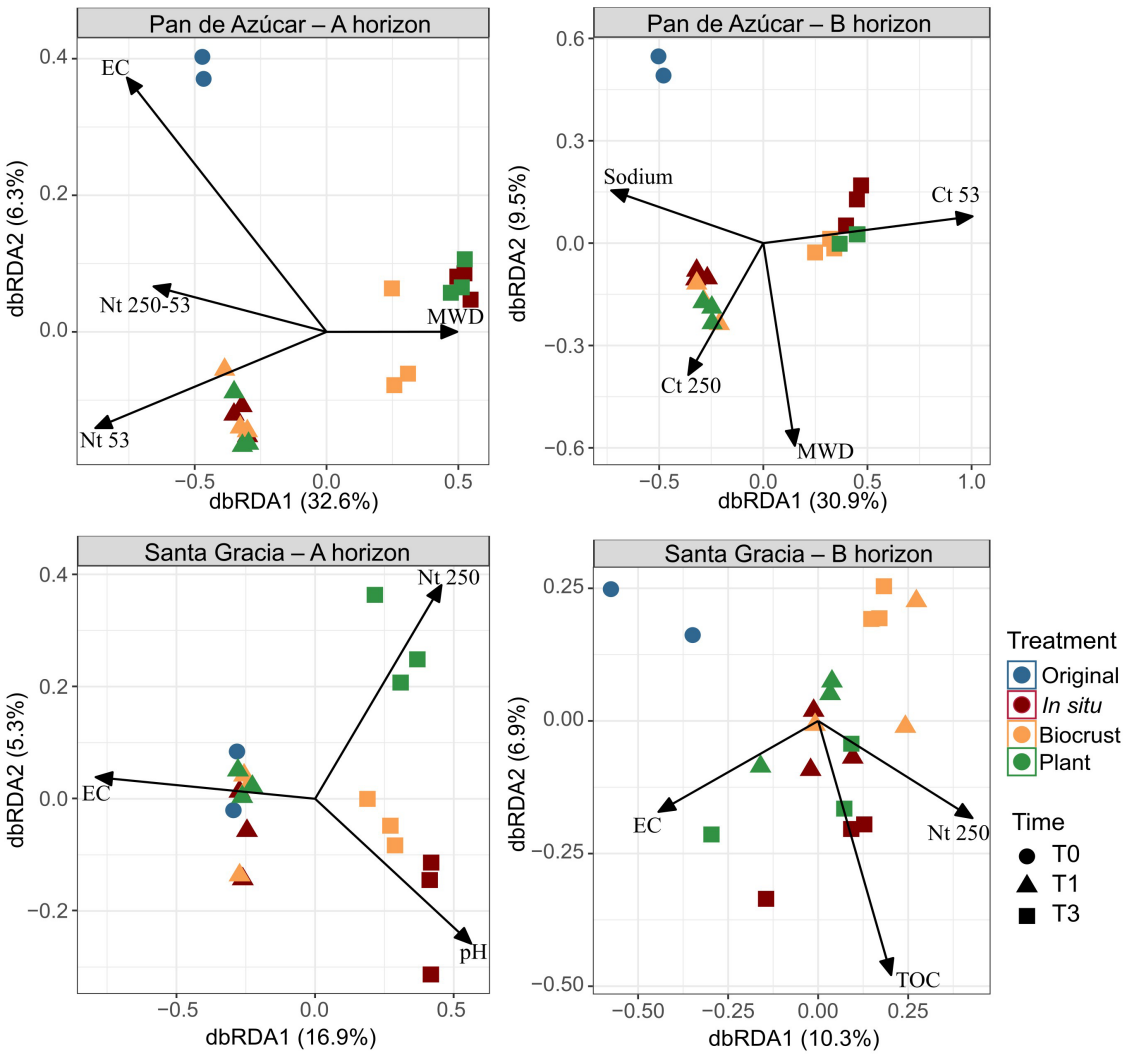
Distance-based redundancy analysis (dbRDA) including soil properties for Pan de Azúcar – A horizon, Pan de Azúcar – B horizon, Santa Gracia – A horizon, and Santa Gracia – B horizon. Significant physicochemical properties included total organic carbon (TOC), nitrogen in macroaggregates (N_t_ 250), nitrogen in large microaggregates (N_t_ 250–53), nitrogen in small microaggregates (N_t_ 53), pH, electrical conductivity (EC), sodium, mean weight diameter (MWD), total carbon in macroaggregates (C_t_ 250), total carbon in small microaggregates (C_t_ 53). Time is represented by T0 (original soil sample), T1 (2 weeks), and T3 (16 weeks). Different color represents different treatments, while shapes represent different sampling times. Each point is the mean of biological triplicates.

We employed a dbRDA model to assess the influence of soil parameters on the variation in bacterial composition (Figure 4). In PA – Ahor, the bacterial composition showed positive correlations with EC at T0, N_t_ in microaggregates at T1, and MWD at T3, collectively explaining 38.9% of the total variation. At T3, differentiation between treatments became apparent. PA – Bhor exhibited positive associations with sodium at T0, C_t_ in macroaggregates at T1, and C_t_ in small microaggregates at T3, explaining 40.4% of the total variation. In SG – Ahor, higher EC at T0 and T1 displayed a positive association, while treatments began to differentiate at T3. Notably, a distinct positive trend was observed between N_t_ in macroaggregates and the plant treatment, while *in situ* and BSC treatments were linked to higher pH. These variables accounted for 22.2% of the total variation. In SG – Bhor, the variables explained 17.2% of the total variation, and no discernible patterns were found to differentiate between times or treatments regarding N_t_ in macroaggregates, TOC, or EC.

### 3.5 Change in bacterial community composition

**Figure 5:**
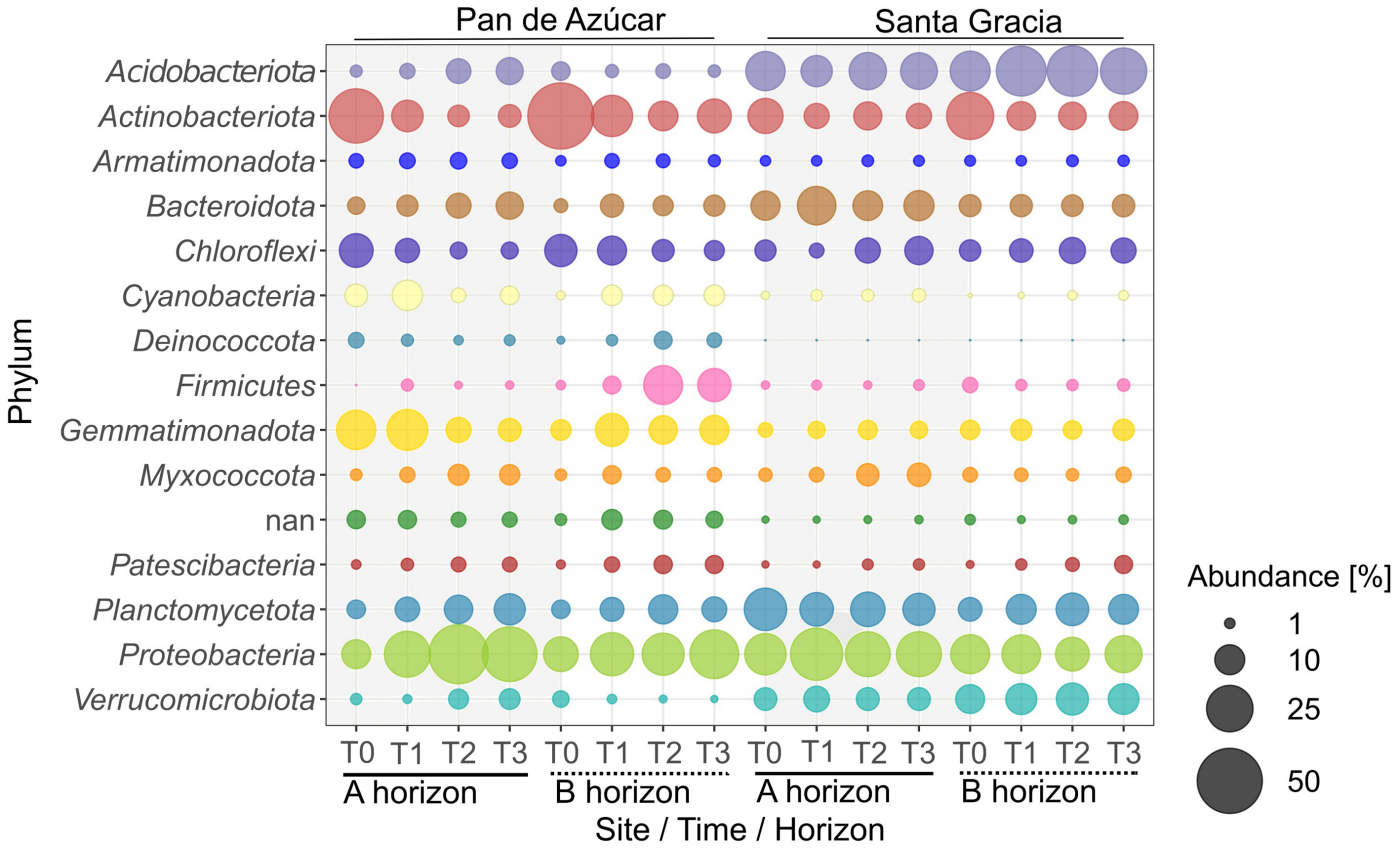
Relative abundance of top 15 bacterial phyla for Pan de Azúcar and Santa Gracia in four-time points of the soil manipulation experiment. Time is represented as T0 (original), T1 (2 weeks), T2 (12 weeks), and T3 (16 weeks). Each bubble is the mean of the different treatments (*in situ*, BSCs, and plants) and biological triplicates.

The analysis of relative bacterial abundances per site revealed that *Proteobacteria* (27.2%), *Actinobacteriota* (12.9%), and *Gemmatimonadota* (10.5%) were the most abundant phyla in PA, while *Acidobacteriota* (20.7%), *Proteobacteria* (20.5%), and *Planctomycetota* (11.8%) dominated in SG. The phyla distribution varied significantly between horizons in both sites (Figure 5, Supplementary Table S6). In PA, *Acidobacteriota* and *Proteobacteria* were more abundant in PA – Ahor (5.4 and 31.9%, respectively) than in PA – Bhor (1.9 and 22.6%, respectively), while *Actinobacteriota* and *Firmicutes* dominated the PA – Bhor (16.7 and 10.3%, respectively) compared to PA – Ahor (9.1 and 0.8%, respectively). In SG, *Proteobacteria* and *Bacteroidetes* were more abundant in SG – Ahor (25.8 and 12.2%, respectively) than in SG – Bhor (15.3 and 5.2%, respectively). *Acidobacteriota* and *Verrucomicrobiota* were more abundant in SG – Bhor (27.1 and 10.9%, respectively) than in SG – Ahor (14.3 and 6.2%, respectively). In addition, some taxa became dominant or scarce over time. For instance, a substantial increase was observed in *Proteobacteria* from T0 to T3, rising from 9.4 to 35% in the A horizon and from 13.7 to 27.7% in the B horizon. Moreover, there are increases in *Planctomycetota* and *Bacteroidota* in both horizons. In contrast, *Actinobacteriota* decreased significantly from T0 to T3 in PA – Ahor (34.5 to 5.6%) and PA – Bhor (51.5 to 13.1%). In contrast to PA, in SG – Ahor, the community exhibited variable changes but remained consistently dominated by the three most abundant taxa, *Acidobacteriota* (14.3%), *Proteobacteria* (25.8%), and *Planctomycetota* (13.2%). SG – Bhor is characterized by the dominance of *Acidobacteriota* (27.1%) and *Proteobacteria* (15.3%), which remained stable, while *Actinobacteriota* exhibited a decrease from 25.9 to 9.2%, being replaced by *Planctomycetota*. Finally, no clear trends in bacterial phyla abundance between SG treatments were observed. Still, PA – Ahor showed an increased abundance of *Acidobacteriota* and *Planctomycetota* in plant treatment (12.7 and 15.5%, respectively) compared to *in situ* treatment (5.9 and 6.8%, respectively).

Correlations between the most abundant bacterial phyla and soil properties were observed. *Acidobacteriota* and *Verrucomicrobia* showed a positive correlation with N_t_ in small microaggregates (*R* = 0.7) and a negative correlation with C_t_ in macroaggregates and pH (*R* = −0.8), as well as with sodium and sulfate (*R* < −0.6). Conversely, *Gemmatimonadota* showed a positive correlation with C_t_ in macroaggregates and pH (*R* = 0.5) but a negative correlation with N_t_ in microaggregates and TOC (*R* < −0.6). *Actinobacteriota* positively correlated with sodium and chloride (*R* = 0.6), while *Bacteroidota* correlated with N_t_ in large microaggregates, N_bulk_, and TOC (*R* > 0.6).

### 3.6 Change in potential predicted functions

Nine thousand two hundred zero ASVs were assigned to 37 ecologically relevant functions described in FAPROTAX (Supplementary Table S7). The two most prevalent functions across all microcosms were chemoheterotrophy (electron acceptor is variable) and aerobic chemoheterotrophy (electron acceptor is O_2_), comprising a mean of 48.2 and 30.2% of the taxonomic abundance in PA and SG, respectively. Additionally, PA showed other abundant functions such as nitrate reduction, phototrophy-associated functions (phototrophy, photoautotrophy, photosynthetic cyanobacteria, and oxygenic photoautotrophy), and functions associated with degradation processes (hydrocarbon degradation, aromatic compound degradation, aromatic hydrocarbon degradation, and aliphatic non-methane hydrocarbon degradation), with a mean of 24% of taxonomic abundance. In contrast, SG exhibited nitrate reduction, ureolysis, and fermentation, with a mean taxonomic abundance of 4.5%. Regarding soil horizons, functions associated with degradation processes showed higher taxonomic abundance in PA – Bhor (mean 14.8%) than in PA – Ahor (mean 1.7%). For SG, chemoheterotrophy, aerobic chemoheterotrophy, ureolysis, and fermentation were more abundant in SG – Ahor than SG – Bhor (mean of 43.3 vs. 23.5%).

Over time, the abundance of chemoheterotrophy and aerobic chemoheterotrophy increased in both horizons of PA from T0 to T3 (mean 33.1 to 55.5%), while it decreased in both horizons of SG (mean 34.2 to 25.8%). Additionally, both horizons of PA showed an increase in the abundance of functions associated with degradation processes from a mean of 0.5% in T0 to 10.8% in T3. In contrast, nitrate reduction decreased over time in both horizons of PA (mean 6.2 to 2.2%). Notably, PA – Ahor and PA – Bhor showed different trends for phototrophy-associated functions, decreasing in Ahor and increasing in Bhor from T0 to T3. As for the SG horizons, SG – Ahor showed a slight increase in the abundance of phototrophy-associated functions (mean 0.7 to 1.5%) and N fixation (mean 0.3 to 1.4%) from T0 to T3, while SG – Bhor remained stable over time. No substantial disparities were observed between treatments for both PA and SG.

Correlations between predicted functions and bacterial phyla were identified. *Proteobacteria* showed a positive correlation with aerobic chemoheterotrophy, chemoheterotrophy (R = 0.9), and cellulolysis (R = 0.6), while *Actinobacteriota* were positively correlated with nitrate reduction (*R* = 0.6). *Cyanobacteria* exhibited a strong correlation with phototrophy-associated functions (*R* > 0.8).

### 3.7 Indicator species

**Figure 6:**
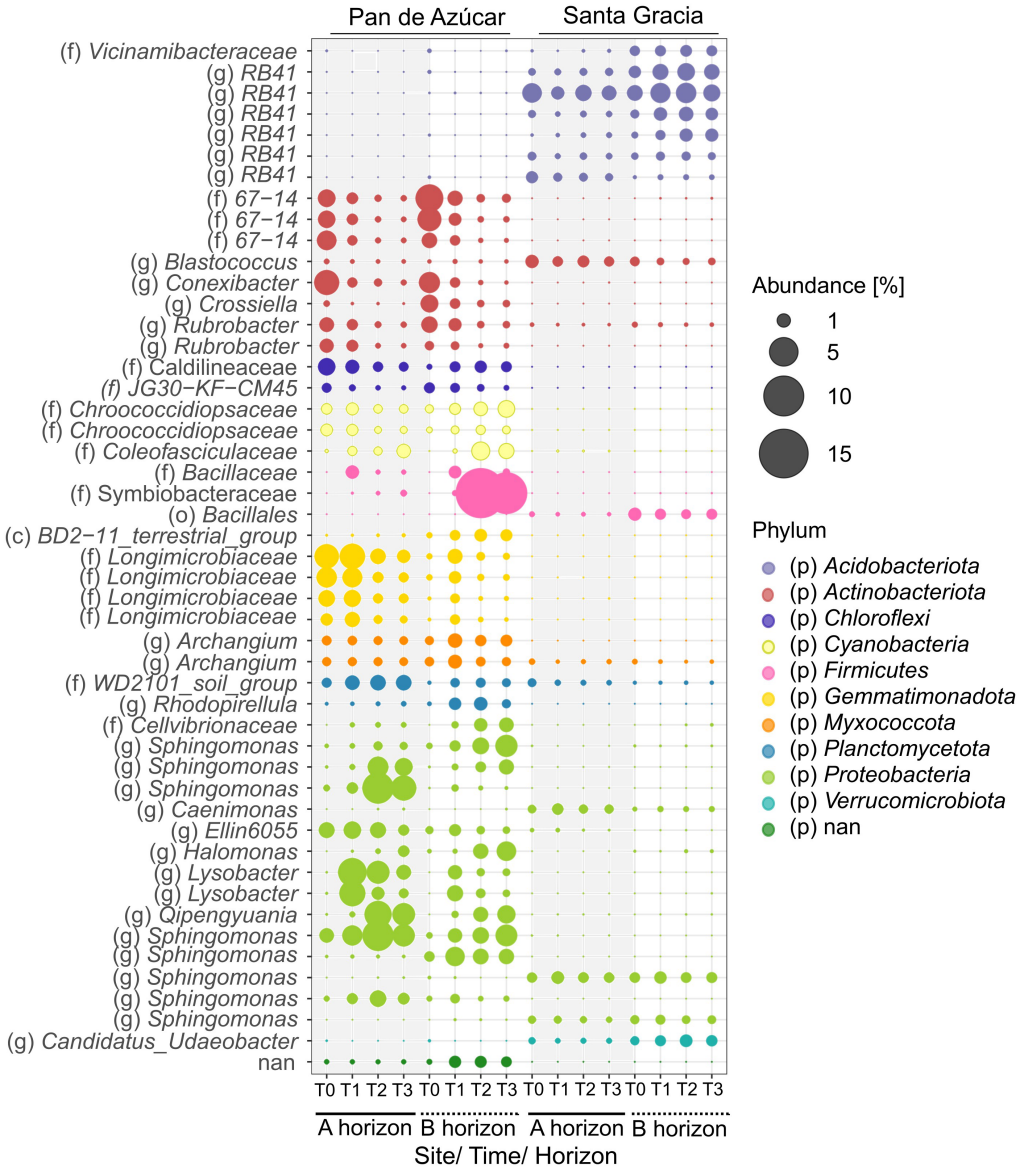
Top 50 relative abundance of indicator species for Pan de Azúcar and Santa Gracia in four time points of the soil manipulation experiment. Colors represented different phyla. Time is represented as T0 (original), T1 (2 weeks), T2 (12 weeks), and T3 (16 weeks). Each bubble is the mean of the different treatments (*in situ*, BSCs, and plants) and biological triplicates. Taxa are shown on the level of class (c), order (o), family (f), and genus (g).

Two hundred eighty ASVs showed a mean over 0.05% and were analyzed for indicator species and the co-occurrence network. Indicator species are species that, by characterizing a group of samples, provide relevant ecological information about the environmental conditions of the ecosystem in which they are found (Dufrene and Legendre, 1997). Using this approach, we explored the association between taxa and a particular site, focusing on temporal changes. Among the analyzed ASVs (280 in total), 163 ASVs exhibited significant associations with one of the sites, with 99 ASVs belonging to PA and 64 ASVs belonging to SG (Figure 6, Supplementary Table S8).

The indicator species belonged to 15 different taxa, with *Proteobacteria* (21 ASVs in PA and 21 ASVs in SG), *Acidobacteriota* (3 ASVs in PA and 26 ASVs in SG), *Actinobacteriota* (15 ASVs in PA and 5 ASVs in SG), *Gemmatimonadota* (14 ASVs in PA and 1 ASV in SG), and *Chloroflexi* (9 ASVs in PA and 1 ASV in SG) being the most abundant, accounting 71% of the total indicators. Notably, the genus *RB41* (12 ASVs), family *Commamonadaceae* (5 ASVs), and order *Vicinamibacterales* (5 ASVs) showed potential as putative indicator taxa in SG, while the family *Longimicrobiacea* (11 ASVs) and the order *Thermomicrobiales* (6 ASVs) were notable indicators in PA. The family *Sphingomonadaceae* was well represented as an indicator species in PA (13 ASVs) and SG (5 ASVs). Regarding soil horizons, 102 ASVs were significantly associated with one of the horizons by site (Supplementary Table S8). The highest abundance of indicator species was observed in PA – Bhor (24.5%), followed by PA – Ahor (13.1%), SG – Bhor (11.3%), and SG – Ahor (5.1%). Among these, the most abundant ASV were *Symbiobacteraceae* (mean 7.8%) in the PA – Bhor, followed by *Sphingomonas* (mean 3.1%) and *Lysobacter* (mean 2.7%) in PA – Ahor. The abundance of indicator species remained stable in SG, while PA fluctuated over time. For instance, indicator ASVs belonging to *Actinobacteriota* (A and B horizons) and *Gemmatimonadota* (A horizon) decreased in abundance over time, while those belonging to *Proteobacteria* showed an increasing trend (Figure 6). No indicator species were identified based on the treatment.

### 3.8 Co-occurrence network

**Figure 7:**
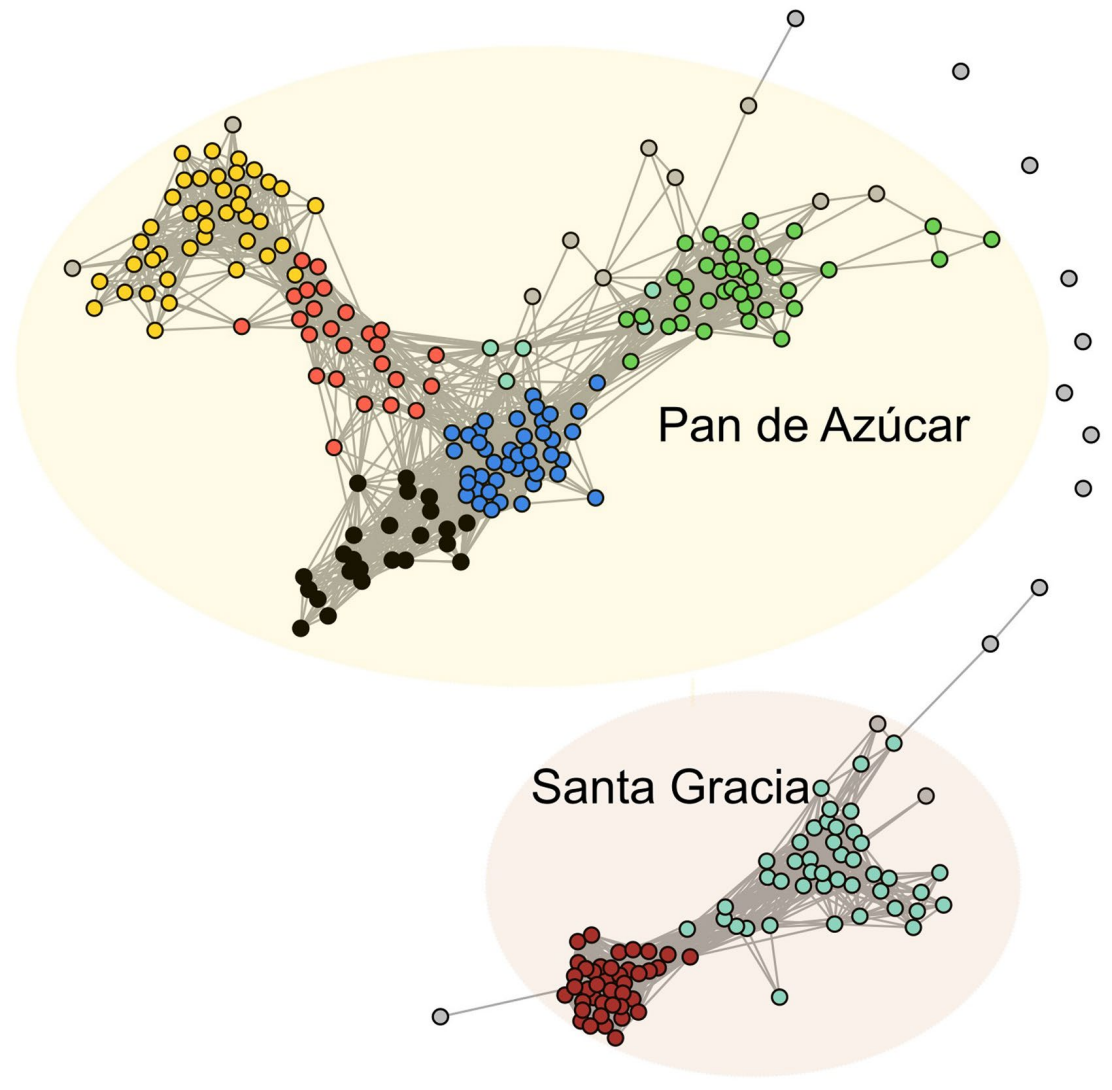
Co-occurrence network of bacterial ASVs. The nodes (ASVs) in the network are colored by modularity. The connections stand for a strong (*R* > 0.7) and significant (*p* < 0.01) correlation. The network was subdivided into two subnetworks corresponding to Pan de Azúcar and Santa Gracia.

We employed a co-occurrence network to explore the dynamics and relationships within bacterial communities across both sites (PA and SG) sharing the same parent material. Among the 280 ASVs analyzed, we identified seven modules of highly correlated ASVs represented by different colors (Figure 7). Based on their correlation patterns, these modules formed two sub-networks: Subnetwork 1 consisted of the brown and turquoise modules, while Subnetwork 2 comprised the green, red, yellow, black, and blue modules. Notably, the ASVs within the subnetworks showed different preferences for specific sites. Subnetwork 1 was predominantly found in SG, whereas Subnetwork 2 was more abundant in PA (Supplementary Figure S5). In subnetwork 1 (SG), *Acidobacteriota* (34), *Proteobacteria* (21), and *Verrucomicrobiota* (14) accounted for 78.4% of the total ASVs. In subnetwork 2 (PA), the most abundant taxa were *Proteobacteria* (47), *Actinobacteriota* (23), and *Gemmatimonadota* (21), representing 49% of total ASVs.

We proceeded to analyze the topological properties of each subnetwork to understand the interrelationship patterns between nodes, as summarized in Table 1. Initially, the network consisted of 273 nodes (ASVs) and 3,203 edges (correlations). After subdivision, the subnetwork 2 (PA) exhibited more nodes and edges, accounting for 66% of all ASVs. Subnetwork 1 (SG) displayed a higher average degree (node connectivity degree), connectance, and clustering coefficient (degree of nodes tended to cluster together), indicating highly connected and densely clustered modules. On the other hand, subnetwork 2 (PA) showed higher modularity (degree of division into modules), indicating a clear division into modules. Additionally, we observed that the abundance patterns of ASVs within the modules varied depending on the horizon. In PA, the green and blue modules were more abundant in the A horizon, while the yellow and red modules were more abundant in the B horizon (Supplementary Figure S5). In SG, the ASVs of the brown module were more abundant in the B horizon, while the turquoise module exhibited a slightly higher abundance in the A horizon.

**Table 1:** Co-occurrence network topological metrics.

Network metrics	Pan de Azúcar	Santa Gracia
No. of nodes^1^	185	88
No. of edges^2^	2,054	1,149
Average degree^3^	11.1	13.02
Cluster coefficient^4^	0.639	0.798
Modularity^5^	0.589	0.429
Connectance^6^	0.060	0.148

1 Number of ASVs with at least one correlation.

2 Number of strong and significant correlations between nodes.

3 How many connections on average each node has to another unique node in the network.

4 Degree to which nodes tend to cluster together.

5 Indicate the partition in modules.

6 Ratio between the number of realized edges and the number of potential edges within the network (Dini-Andreote et al., 2014; Karimi et al., 2017).

We identified the top hub ASVs per module to gain further insights into the highly connected species within the co-occurrence networks. The hub ASVs in SG belonged to the family *Comamonadaceae* in the turquoise module and the genus *Candidatus* Udaeobacter in the brown module. On the other hand, in PA, hub ASVs were identified from the family *Sphingobacteriaceae* (green), *Longimicrobiacea* (blue), *67–14* (black), *Ardenticatenaceae* (red), and *WD2101 soil group* (yellow).

**Figure 8:**
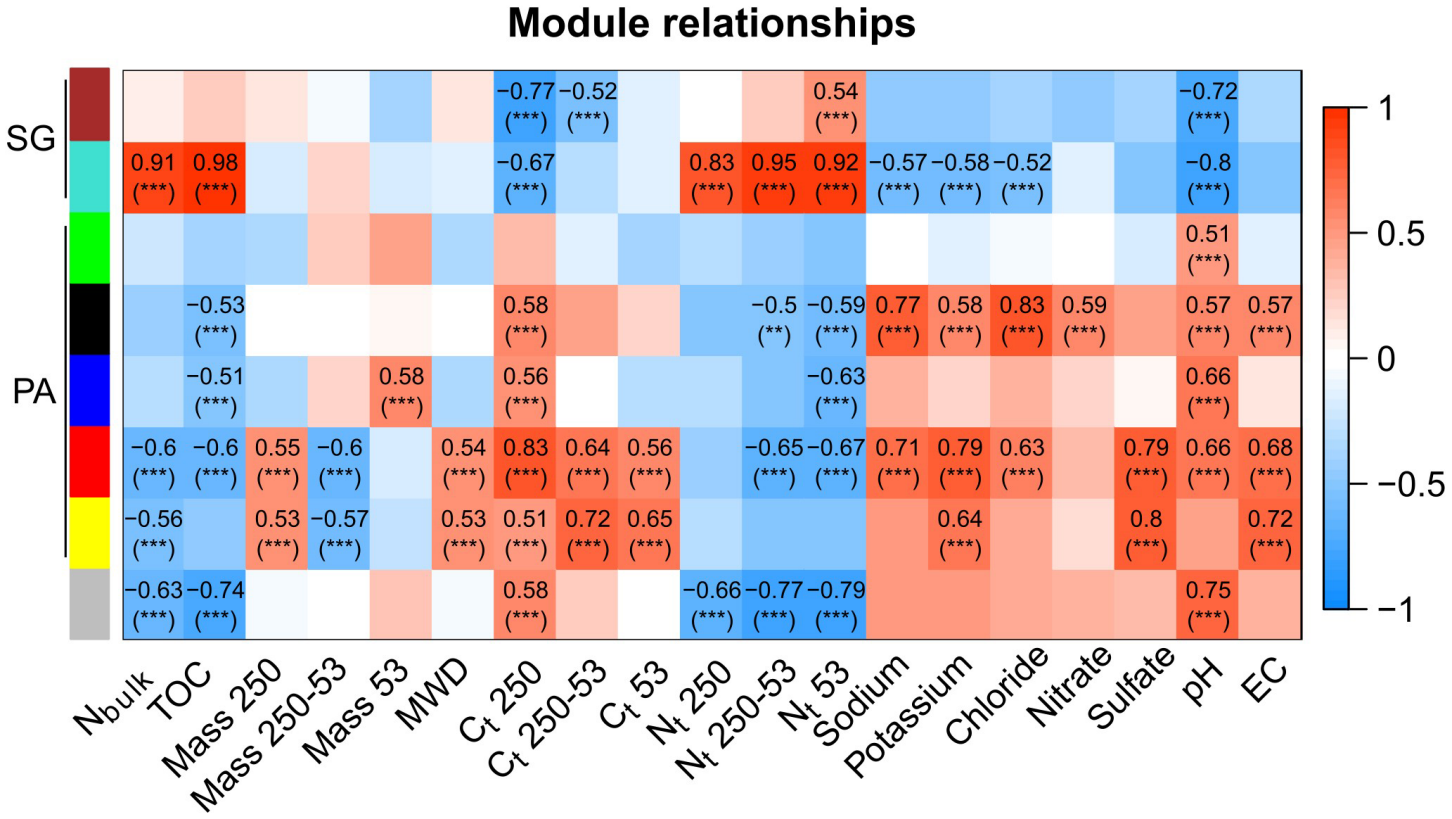
Pearson correlation of modules from the co-occurrence network and soil physicochemical properties in Pan de Azúcar (PA) and Santa Gracia (SG). Only correlations greater than *R* = 0.5 are shown. Significance levels are indicated as follows: “**” for value of *p* < 0.01 and “***” for value of *p* < 0.001. The physicochemical properties included nitrogen in the bulk soil (N_bulk_), total organic carbon (TOC), total mass in macroaggregates (Mass 250), total mass in large microaggregates (Mass 250–53), total mass in small microaggregates (Mass 53), mean weight diameter (MWD), total carbon in macroaggregates (C_t_ 250), total carbon in large microaggregates (C_t_ 250–53), total carbon in small microaggregates (C_t_ 53), nitrogen in macroaggregates (N_t_ 250), nitrogen in large microaggregates (N_t_ 250–53), nitrogen in small microaggregates (N_t_ 53), sodium, potassium, chloride, nitrate, sulfate, pH, and electrical conductivity (EC).

We utilized the identified modules to correlate them with physicochemical properties and examine their associations (Figure 8). The modules representing SG displayed a distinct pattern based on organic matter content. The turquoise module positively correlated with TOC, N_bulk_, and N_t_ in different aggregate sizes (*R* > 0.8). Conversely, the turquoise and brown modules negatively correlated with pH (*R* < 0.7). In PA, the black module demonstrated a strong positive correlation with sodium and chloride, while the blue module was correlated with pH (*R* = 0.7). The red module displayed associations with C_t_ content in macroaggregates, sodium, potassium, sulfate, pH, and EC (*R* > 0.7). Additionally, the yellow module exhibited a strong positive correlation with C_t_ in microaggregates, sulfate, and EC (*R* > 0.7).

## 4 Discussion

This study investigated the interplay between biotic and abiotic factors during soil formation within initial arid and semiarid soils under simulated climate change (humid climate conditions). We focused on exploring the direct impact of microbial communities on the early stages of soil formation and their interactions with BSCs and plants by analyzing changes in soil physicochemical properties along the different treatments. Further, we examined the bacterial community response of arid and semiarid soils to simulated climate change to gain insights into their potential adaption to the changed conditions.

### 4.1 Abiotic versus biotic influence on soil formation in arid and semiarid soils under climate change

A simulated humid climate that mimicked a climate change scenario along the Chilean Coastal Cordillera stimulated the initial soil formation in all treatments. The extent of this “climate change” effect on soil formation varied by soil origin and horizon but did not differ from the sterile treatment. This indicates that the abiotic influence on initial soil formation was larger than the biotic influence over the short simulation period. The formation of macroaggregates, the loss of large microaggregates, and the associated increase in MWD in the B horizon of PA were likely influenced by elevated levels of carbonates (e.g., CaCO_3_). The prevalence of inorganic carbon in the form of carbonates is supported by the high C_t_ values coupled with low TOC concentrations, as corroborated by prior studies (Schulze-Makuch et al., 2018; Riveras-Muñoz et al., 2022). Water likely facilitated the diffusion of cations, stimulating carbonate precipitation to form secondary carbonate coatings and bind primary soil particles, thus enhancing soil structure (Bronick and Lal, 2005). Additionally, notable reductions in EC in the A horizon of PA and sodium and chloride concentrations in the B horizon of PA were observed, likely due to the leaching of exchangeable base cations and soluble salts as reported by Bernhard et al. (2018), consistent with previous findings in saline-alkali soils (Wang et al., 2017). Lastly, the increase in TOC in the B horizon of SG compared to the A horizon indicates a contrasting response in carbon mobility and retention between the two horizons, potentially driven by enhanced mineral adsorption capacity or increased occlusion of organic matter into aggregates (Kramer and Chadwick, 2018). These findings highlight the influence of soil origin (site and horizon) and their physicochemical properties on the response to climate change over time.

The microorganisms-plant treatment positively influences arid and semiarid soil stabilization, indicated by increased macroaggregate formation and MWD in SG and the A horizon of PA at 16 weeks relative to the other treatments. In contrast, this beneficial effect was not observed in the B horizon of PA, due to poor root development and premature plant mortality promoted by high salinity and nutrient deficiency during the manipulation experiment. The observed effect can be attributed to the enmeshment and realignment of soil particles by roots and the release of exudates, leading to significant physical, chemical, and biological alterations that influence soil aggregation (Bronick and Lal, 2005). Prior studies support the role of fine sand and fine roots as co-drivers of aggregate stability in early successional stages of mediterranean soils (Erktan et al., 2016) and the promotion of macroaggregate formation through root exudates favoring fungi in carbon-poor forest subsoils (Baumert et al., 2018). Additionally, field experiments have shown that water addition in the presence of plants enhances macroaggregation, probably due to increased root activity, fungal enmeshment, and microbial access to carbon substrates for exopolysaccharide production (Blankinship et al., 2016). While converting desert soil into irrigated cropland with profitable harvest typically requires at least 50 years (Su et al., 2010), and natural soil development processes occur over much longer timescales spanning hundreds to thousands of years (Jenny, 1994), our findings reveal the early measurable impact of microorganisms-plant associations on the soil stabilization even in short-time scales of weeks.

Microorganisms alone and in conjunction with BSCs had no discernible impact on the measured physicochemical parameters compared to sterile soils under the experimental settings. The absence of changes can be attributed to the potentially more prominent influence of water, which likely overshadowed the effects of microorganisms, resulting in a water-masking effect. This finding aligns with a previous six-month manipulation experiment on mediterranean soil, highlighting the increased significance of microorganisms in aggregation under drier conditions, where limited water availability restricts abiotic macroaggregate formation but supports active microbial communities (Blankinship et al., 2016). Similarly, a field study proposed that plants and organic matter exert a stronger influence on aggregation than BSCs in wetter conditions along a climate gradient, while BSCs are significant compared to bare soil, including microorganisms in drier environments (Riveras-Muñoz et al., 2022). Moreover, a previous study in SG reported no significant increase in N-fixation and even a decrease in nitrogen mineralization in SG when soil moisture reached 57% of water-filled pore space (Seuss et al., 2022). Based on these findings, our results suggest that the applied water content (65% water-filled pore space) may exceed the threshold required to activate specific biogeochemical processes facilitated by microorganisms. Alternatively, it is plausible that soil properties like organic carbon and acidity exhibit slower changes than the microbial community structure alone, considering their short generation times, allowing them to adapt and respond rapidly to environmental changes (Placella et al., 2012).

On the other hand, our study may have overlooked the initial effects of microorganisms on soil properties when analyzing bulk soil samples taken across an entire soil horizon. This limitation could also dilute the impact of BSCs, which primarily influence the soil properties near the surface, progressively decreasing with depth (Miralles et al., 2020). Microorganisms might influence soil properties at a smaller spatial scale beyond the resolution of our bulk soil analyses. For instance, nanoscale secondary ion mass spectrometry analysis in vertisol and alfisol soils demonstrated the crucial role of microbially metabolized nitrogen in promoting organo-mineral associations, impacting organic matter cycling, immobilization, and storage (Kopittke et al., 2018). However, despite the lack of independent impact detected, microorganisms likely played an important role in improving soil conditions by mitigating abiotic stress factors, such as high pH, salinity, and nutrient limitations for plant-microorganism treatment (Etesami and Beattie, 2017). This beneficial effect is exemplified by the active recruitment of growth-promoting bacteria, including N-fixing bacteria, by plant roots in the Atacama desert (Eshel et al., 2021), which may have influenced the observed differentiation of bacterial communities among treatments by the end of the soil manipulation experiment.

### 4.2 Bacterial communities inhabiting arid and semiarid soils respond differently to climate change

Differential responses of bacterial communities were observed at the two different sites under simulated climate change. Notably, bacterial communities increased in gene copy numbers in PA, the arid site (in contrast to SG, the semiarid site), revealing higher bacterial activity or growth based on higher cellular ribosome contents (Placella et al., 2012). This increase in gene copy numbers can be attributed to the “Birch effect,” which is influenced by wetting and promotes the breakdown of aggregates, mineralization of dead microbial cells and osmolytes, and subsequent release of nutrients such as carbon (Jarvis et al., 2007). This phenomenon has been observed in arid and semiarid soils (Armstrong et al., 2016; Seuss et al., 2022), where releasing organic compounds potentially enhances bacterial reproduction and growth. This finding could explain the correlation between the bacterial community and nitrogen content in the A horizon of PA after two weeks of the soil manipulation experiment or the enhanced predicted functions related to chemoheterotrophy and organic matter degradation processes. These results evidence alterations in substrate availability and concomitant growth dynamics, which can be linked to the initial activation of soils triggered by nutrient inputs in the system promoted by humid climate conditions (Nemergut et al., 2016; Chen Y. et al., 2021).

Contrasting the gene copy numbers, changes in diversity indices over time reveal a decrease in the Shannon index in PA while remaining stable in SG. This is attributed to the degree of bacterial specialization, which is different in both sites. PA exhibits a high degree of bacterial specialization as an evolutionary response to a stable environment in time and space (Rodriguez et al., 2022), while sudden climate shifts can act as stressors that promote a reduction in diversity (Clavel et al., 2011). In contrast, SG represents a heterogeneous and disturbed ecosystem dominated by bacterial generalists (Rodriguez et al., 2022), benefiting from their metabolic flexibility to thrive in highly dynamic environments (Hawkes et al., 2020; Chen Y.-J. et al., 2021). Our findings follow a previous study demonstrating that frequent drying-wetting stress may favor tolerant microbial taxa in SG (Seuss et al., 2022). This also can be illustrated by other studies where in the hyperarid Atacama Desert, diversity decreases when water is rapidly and abundantly applied (Azua-Bustos et al., 2018), while a desert steppe with mean annual precipitation of 289 mm showed no response to altered precipitation regimes (Na et al., 2019). Furthermore, Stovicek et al. (2017) found higher diversity in deserts due to disconnected soil niches, but wetter soil conditions reduce diversity through dispersion, connectivity, homogenization, and nutrient fluxes. Given the association between bacterial diversity and enzymatic activities related to nitrogen and phosphorus cycles, as well as plant productivity (Delgado-Baquerizo et al., 2016; Luo et al., 2018), the decrease of diversity in PA could negatively affect soil formation due to a smaller number of available metabolic pathways (Shen et al., 2023).

The degree of bacterial specialization is reflected in the shift of taxonomic composition observed at different sites. PA exhibits an early community reassembly from phyla to the ASV level, along with variations in indicator species, indicating a different tolerance threshold compared to SG. In the A horizon of SG, the dominant phyla and indicator species remained steady, but a change occurred at the ASV level after 16 weeks, while the B horizon maintained ASV stability from 2 to 16 weeks. These findings can potentially be attributed to two microbial adaptation strategies (Uritskiy et al., 2019): In PA, new bacteria from the seed bank come in through niche intrusion, while SG adjusts its existing fitness-based community structure to withstand new selective pressures and prevents the dominance of new bacteria. A study on grasslands assessing drought and precipitation extremes based on mean annual precipitation revealed an unchanged soil bacterial composition, suggesting a high level of community and functional resistance in areas with climate variability (Hawkes et al., 2020). Conversely, studies in the Namib and Negev deserts demonstrate changes in bacterial composition following precipitation events (Armstrong et al., 2016; Stovicek et al., 2017; Ouyang and Li, 2020), supporting the notion that more arid ecosystems are more susceptible to precipitation impacts.

The succession dynamic in PA is evident through the shift in taxonomic composition, initially dominated by *Actinobacteriota* and gradually replaced by *Proteobacteria*. *Actinobacteriota* thrive in oligotrophic environments, particularly during early soil succession, and can affect the soil organic matter formation and biogeochemical cycles (Zhang et al., 2019; Yu et al., 2022). However, their abundance decreases with increasing water availability as the corresponding species are adapted to dry conditions (Barnard et al., 2013; Maestre et al., 2015; Stovicek et al., 2017). This shift is exemplified by the reduction in desiccation-resistant species like *Rubrobacter* or *Solirubrobacterales* (*Conexibacter* and *67–14*), which include genes for chemosynthetic CO_2_ fixation (Schulze-Makuch et al., 2018; Meier et al., 2019). Similarly, *Gemmatimonadota*, known for its potential roles in carbon fixation and N_2_O reduction and adapted to extreme environments such as saline soils, also showed a decreasing abundance with increased water content (Mujakić et al., 2022). Notably, the *Longimicrobiaceae* family within this phylum, which could facilitate phosphate solubilization in the soil, showed a marked decline (Korkar et al., 2022). In contrast, while *Cyanobacteria* overall declined, the *Chroococcidiopsaceae* genus, a potential primary producer in the Atacama Desert using nitrate and ammonium, remains stable (Schulze-Makuch et al., 2021). Our study aligns with previous research showing the potential ability of PA bacterial communities to fix and accumulate organic matter (Rodriguez et al., 2022), highlighting the potential negative consequences of diminishing these bacterial taxa on soil fertility.

Increased water availability, the release of organic compounds, and the accumulation of elements via long-term weathering and climate processes can create a relatively nutrient-rich habitat, favoring copiotroph groups such as *Bacteroidota* (correlated with nitrogen and TOC) and *Proteobacteria* (correlated with chemoheterotrophy) in PA (Na et al., 2019; Zhao et al., 2019). Among these, *Proteobacteria* are widely distributed and preferentially use the labile pool of organic carbon (Fierer et al., 2007). They exhibit metabolic versatility, encompassing functional groups such as phototrophs, chemoorganotrophs, and chemolithotrophs (Kersters et al., 2006), allowing them to utilize multiple energy sources, carbon substrates, and electron acceptors (Chen Y.-J. et al., 2021). From this phylum, functions such as microaggregate formation through the excretion of extracellular polymeric substances have been associated with the genus *Sphingomonas* and also with the family *Sphingobacteraceae* (*Bacteroidota*), which include several indicators and one hub species (Caesar-TonThat et al., 2007; Pankratov et al., 2007; Vuko et al., 2020). Remarkably, their abundance increased over time, potentially influencing the stabilization of soil aggregates and the increased MWD in our manipulation experiment. While the new community displays increased versatility and potential new functions, it is crucial to recognize that the loss of taxa could disrupt soil ecosystem processes, functions, and stability (Bestion et al., 2020).

*Proteobacteria*, *Acidobacteriota*, and *Planctomycetota* steadily dominated the A horizon of SG and the B horizon of SG at 16 weeks. This finding is consistent with previous studies reporting a prevalence of these phyla during the successional development of tundra soils, the desert, and after deglaciation (Zhelezova et al., 2019; Yu et al., 2022; Shen et al., 2023). *Acidobacteriota*, known as heterotrophic-oligotrophs, are well-adapted to harsh environmental conditions with limited carbon availability (Fierer et al., 2007; Zhao et al., 2019). Genomic analysis of *Acidobacteriota* has revealed their metabolic flexibility and versatility, with the potential capacity to participate in sulfur metabolism and utilize diverse carbohydrates, as is also indicated by the indicator species *RB41* (Eichorst et al., 2018; Kalam et al., 2020; Stone et al., 2021). Furthermore, *Acidobacteriota* has numerous genes related to the nitrogen cycle (Eichorst et al., 2018), highlighting their potential role in nitrogen dynamics, further supported by its correlation with N_t_. On the other hand, *Planctomycetota* exhibits both copiotrophic and oligotrophic features, adapting to varying carbon availability (Lauro et al., 2009). The metabolic versatility of these dominant phyla positions them as potential players in organic matter balance and ecological dynamics.

Our co-occurrence network analysis confirms the distinction between the soil environments of PA and SG. Specifically, the SG subnetwork demonstrates greater complexity than PA, as indicated by a higher average degree, connectance, and lower modularity (Karimi et al., 2017; Tu et al., 2020). This finding is in agreement with previous research that has reported a higher average degree in more advanced successional stages within an early chronosequence (Sun et al., 2017), in conjunction with increased nutrient availability (Zhao et al., 2019) and with higher diversity (Tu et al., 2020), as found in SG. Furthermore, our findings indicate a higher resilience to environmental perturbations in SG, consistent with previous studies associating higher connectance and complexity to functional redundancy of interactions (Tylianakis et al., 2010; Karimi et al., 2017; Wu et al., 2021). On the other hand, the PA subnetwork displayed a more modular structure, indicating that bacterial communities within the same module share a similar ecological niche (Tu et al., 2020). These niches are shaped by nutritional preferences, functional distinctiveness, and interaction with physicochemical properties (Banerjee et al., 2016). Specifically, the red and yellow modules, mainly composed of *Proteobacteria*, showed associations with C_t_ in macro- and large microaggregates and EC, suggesting their involvement in carbon sequestration as carbonates in saline-alkali soils (Qu et al., 2022). This, in turn, could link the carbonate content and MWD in these modules. Additionally, the yellow module, positively correlated with sulfate, contained the species *MW2101* (phylum *Planctomycetota*), among others encoding proteins similar to sulfatases, potentially enabling efficient access to the carbon of sulfated compounds as energy and carbon sources (Dedysh et al., 2021). In SG, the turquoise module positively correlated with nitrogen, which could be related to the high number of *Acidobacteriota* and the N-fixing family *Comamonadaceae* in this module (Eichorst et al., 2018; Vuko et al., 2020). Our results highlight the importance of bacterial clustering within specific modules, providing information about niche partitioning, synergistic relationships, and overall soil functioning (Dini-Andreote et al., 2014).

### 4.3 Soil and climate legacy play a role in shaping the response of initial soil formation and bacterial communities to climate change

Under the simulated climate change scenario, the arid soil of PA exhibited a more pronounced response compared to the semiarid soil of SG. The greater shifts in physicochemical properties and soil bacterial communities in arid soils under climate change are attributed to the influence of climate legacies, which have emerged as key drivers shaping the response of bacterial communities to climate disturbances (Delgado-Baquerizo et al., 2017a, 2018). Evidence from cosmogenic nuclide analysis supports the existence of a stable and hyperarid climate in PA throughout the Pliocene and Pleistocene (Placzek et al., 2009). Even other studies indicated climate stability from the late Jurassic, as inferred from paleomagnetic data (Hartley et al., 2005). In contrast, the region between 30° to 35° S with the study site SG has experienced significant influences from glacial advances, periglacial effects, and climatic changes (Arroyo et al., 2012). The differences between both sites and our findings indicate that SG harbors a more tolerant bacterial community, enabling them to adopt different functional repertoire modes, compete for resources, and maintain taxonomic stability even under changing environmental conditions. In contrast, being sensitive and reactive, the bacterial community in PA has changed toward a more versatile community with potentially new functions, as can be inferred from the new taxa in response to the humid climate of Nahuelbuta that has been applied experimentally to mimic climate change. However, this bacterial replacement, associated with vulnerability to disturbances, can impact the ability of the community to self-sustain its functioning and ecosystem stability (Chen Y. et al., 2021). Therefore, it is necessary to investigate whether taxonomic changes are linked to functional changes using additional tools such as metagenomics and metatranscriptomics to gain insights into the potential roles of bacteria in the initial soil transformation.

We further propose that soil and climate legacy is important not only for the soil bacterial community and its response but also for future soil development under climate change. Soil and climate legacies, which can be understood as the resilience of the system, have influenced less soil development in PA (as indicated by high pH and salinity values (EC) and low TOC, nitrogen, bacterial abundance, and plant development). In contrast, SG has shown a progressive soil formation with organic matter accumulation, increased vegetation cover (30–40% shrubs and cacti), and a pH decrease due to increased precipitation and reduced temperature from PA to SG (Bernhard et al., 2018). This presumably promoted different short-term trajectories of soil formation between sites and horizons, impacting the decomposition rate of organic matter, mineral leaching, and plant development differently. Previous studies support our findings by studying the legacy effects of climate and soil moisture on carbon stocks, nutrients, plant growth, and mycorrhizal responsiveness (Cavagnaro, 2016; Delgado-Baquerizo et al., 2017b). In turn, the legacy of resource availability modulates microbial resource responses (Yuan et al., 2022). Therefore, the soil and climatic legacy shapes soil formation by influencing microbial communities, physicochemical properties, biogeochemical cycles, nutrient availability, and carbon dynamics, providing crucial insights for managing soil sustainability in different soil environments.

## 5 Conclusion

Soil formation is crucial for life on Earth and biogeochemical cycles; therefore, understanding the role of microorganisms in this process is vital for gaining insights into ecosystem dynamics. Our findings demonstrate that microorganism-plant interaction drives short-term soil development and stabilization in arid soils exposed to humid climate conditions, even on short time scales of weeks. Interestingly, microorganisms alone and in conjunction with BSCs did not play a measurable role in soil formation under the same conditions. Hence, the microorganism-plant interaction has a faster and more robust impact on altering soil physicochemical properties in humid climate conditions than microorganisms alone. Furthermore, we identified a distinct sensitive and specialized bacterial community in arid soil, contrasting with the more complex, versatile, and stable bacterial community observed in semiarid soil, which we attribute to climate legacies. Our findings are relevant to understanding the intricate dynamics and complexity of bio-geo interactions between biota and climate on soil formation, providing insights into soil management (e.g., efficient irrigation), long-term ecosystem sustainability in arid regions, and the bacterial response to climate change.

## Data availability statement

The datasets presented in this study can be found in online repositories. The names of the repository/repositories and accession number(s) can be found below: EBI – https://www.ebi.ac.uk/ena/browser/view/PRJEB60029.

## Author contributions

VR: Formal analysis, Investigation, Methodology, Visualization, Writing – original draft, Writing – review & editing. AB: Data curation, Formal analysis, Methodology, Supervision, Writing – review & editing. KW: Formal analysis, Methodology, Writing – review & editing. NR-M: Formal analysis, Methodology, Writing – review & editing. RO: Conceptualization, Supervision, Writing – review & editing. SL: Supervision, Writing – original draft, Writing – review & editing. JK: Formal analysis, Methodology, Writing – review & editing. OR: Formal analysis, Methodology, Writing – review & editing. CM: Conceptualization, Writing – review & editing. OS: Conceptualization, Writing – review & editing. TS: Conceptualization, Writing – review & editing. DW: Conceptualization, Supervision, Writing – original draft, Writing – review & editing.
