# Supplementary material for: Microbial impact on initial soil formation in arid and semiarid environments under simulated climate change
Source: Front Microbiol. 2024 Jan 17;15:1319997. doi: 10.3389/fmicb.2024.1319997 (PMC10827993; doi:10.3389/fmicb.2024.1319997)
Supplement: Supplementary file 2 [file Data_Sheet_1.docx]

Supplementary Material


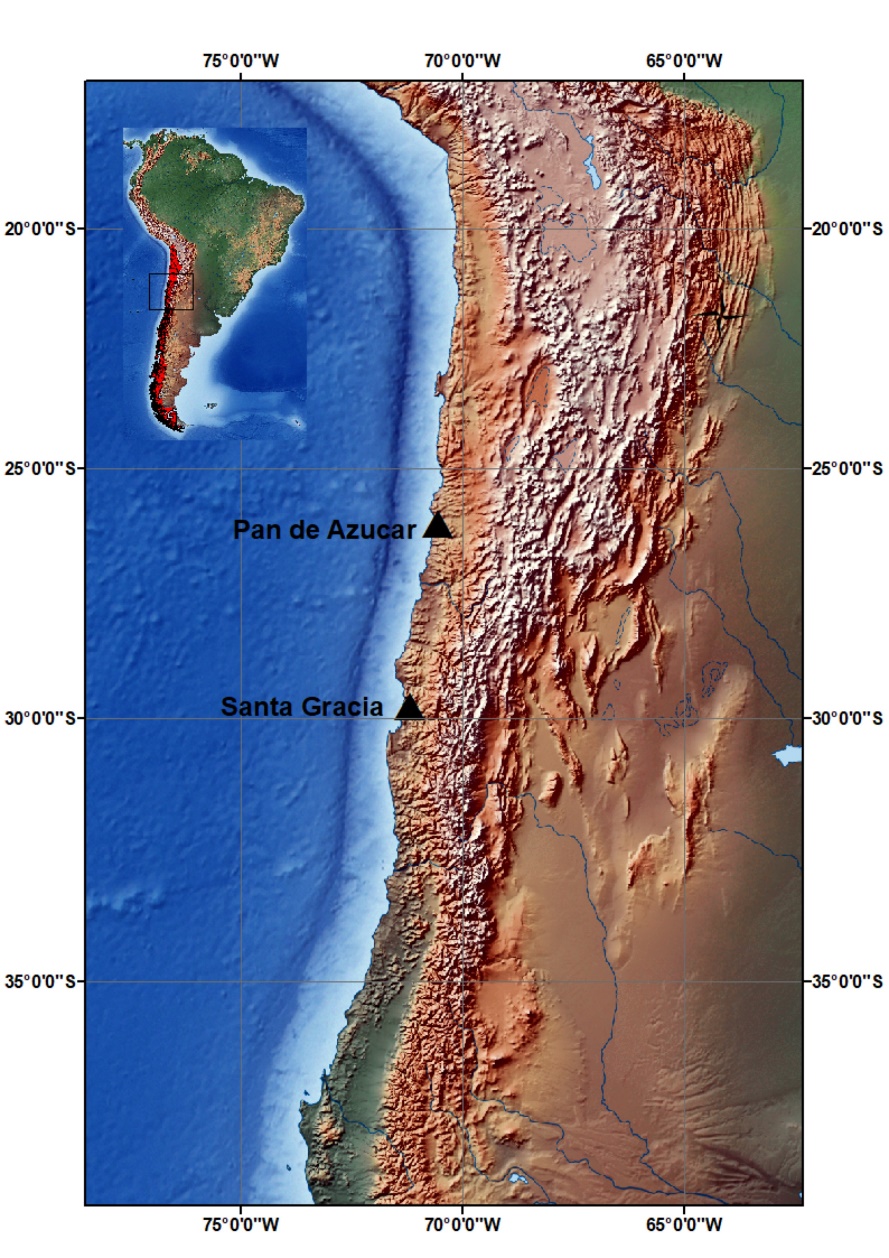


Figure S1. Location of the two study sites of Pan de Azúcar and Santa Gracia in the Chilean Coastal Cordillera.


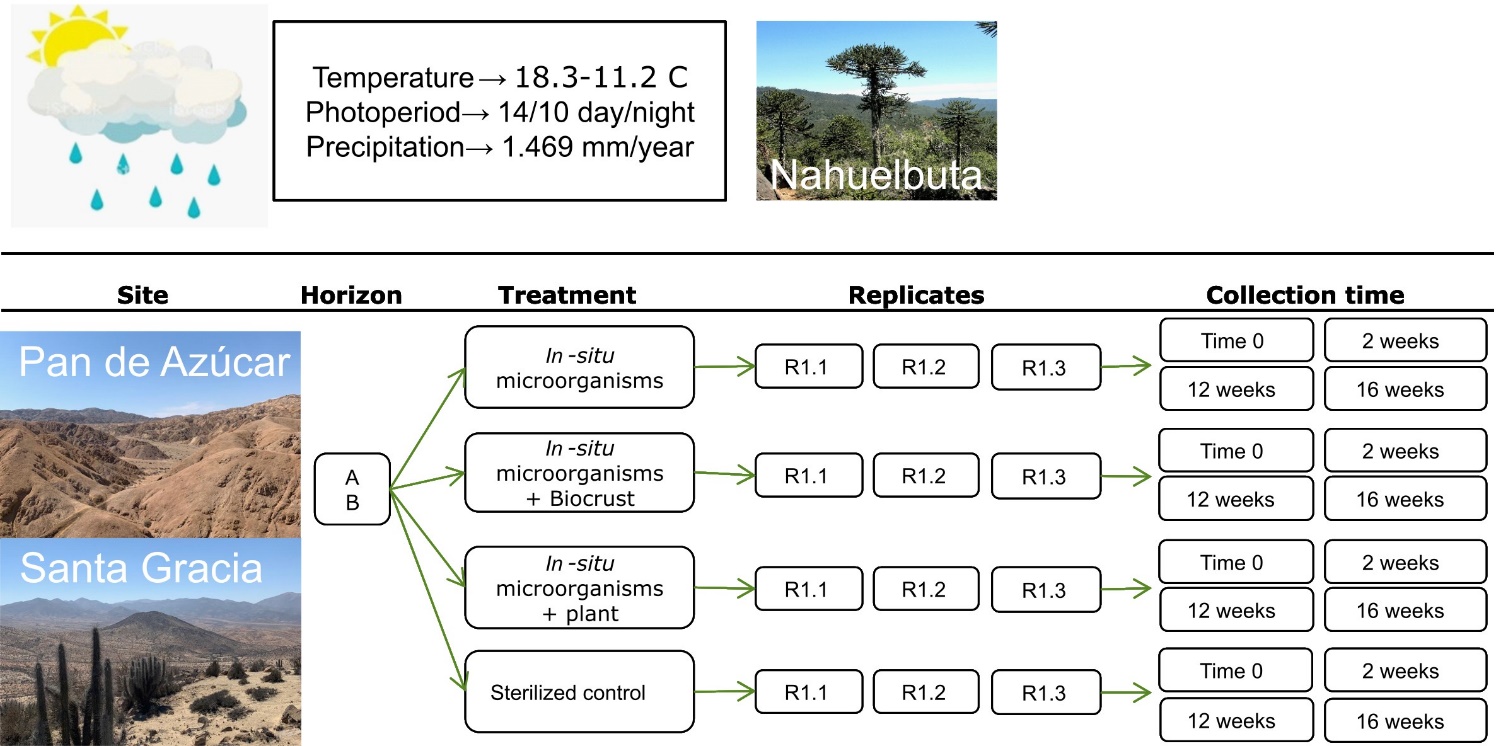


Figure S2. Soil manipulation experiment setup.


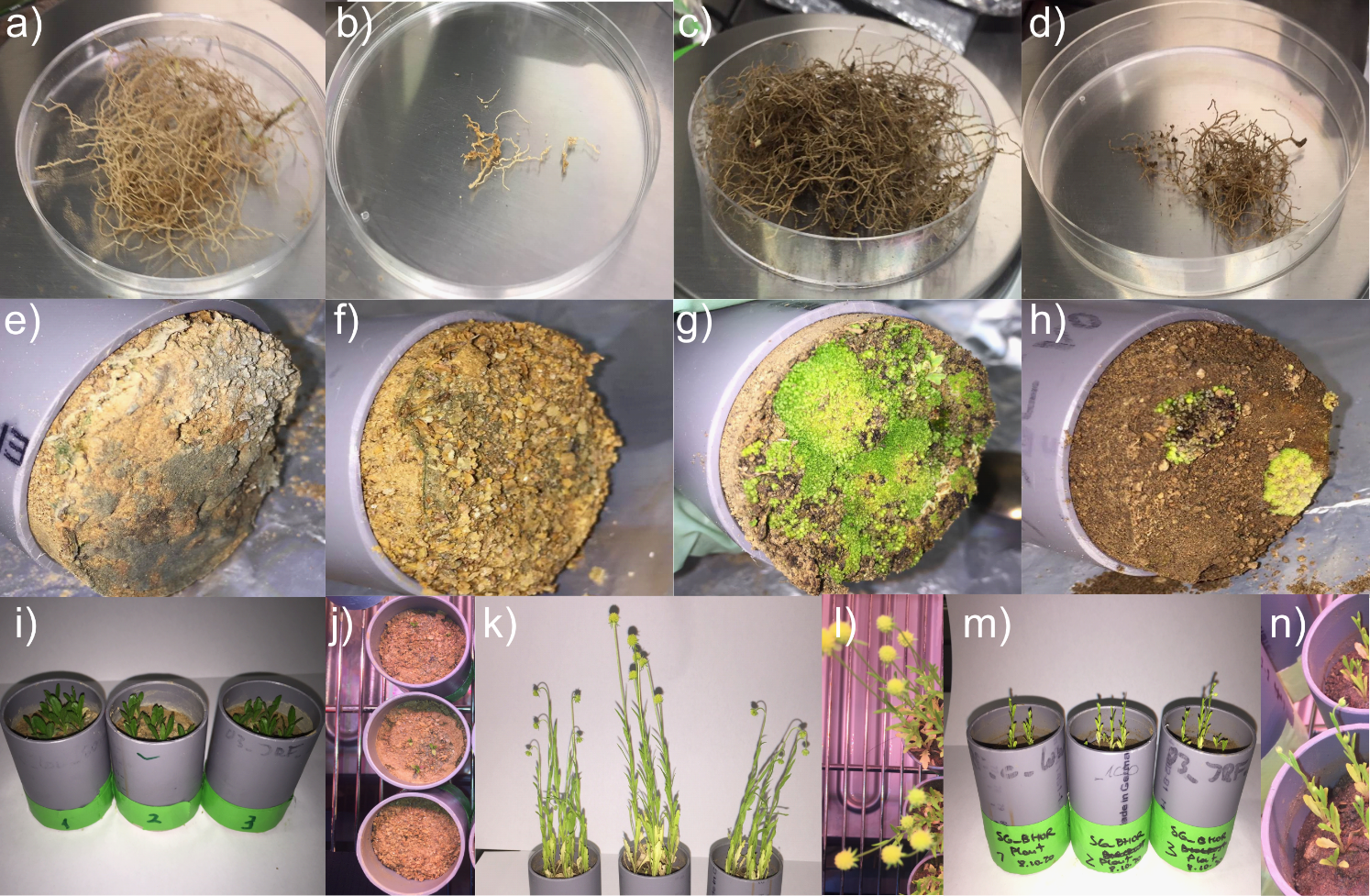


**Figure S3.** Detailed Microcosms of Pan de Azúcar and Santa Gracia after 12 weeks of incubation. a) Roots in A horizon of Pan de Azúcar, b) Roots in the B horizon of Pan de Azúcar, c) Roots in the A horizon of Santa Gracia, d) Roots in the B horizon of SG, e) Biological soil crust in the A horizon of Pan de Azúcar, f) Biological soil crust in the B horizon of Pan de Azúcar, g) Biological soil crust in the A horizon of Santa Gracia, h) Biological soil crust in the B horizon of SG, i) Plants in the A horizon of Pan de Azúcar, j) Plants in the B horizon of Pan de Azúcar, k) Plants in the A horizon of Santa Gracia, l) Flowers in the A horizon of Santa Gracia, m) Plants in the B horizon of Santa Gracia, n) Flower buds in the B horizon of Santa Gracia.


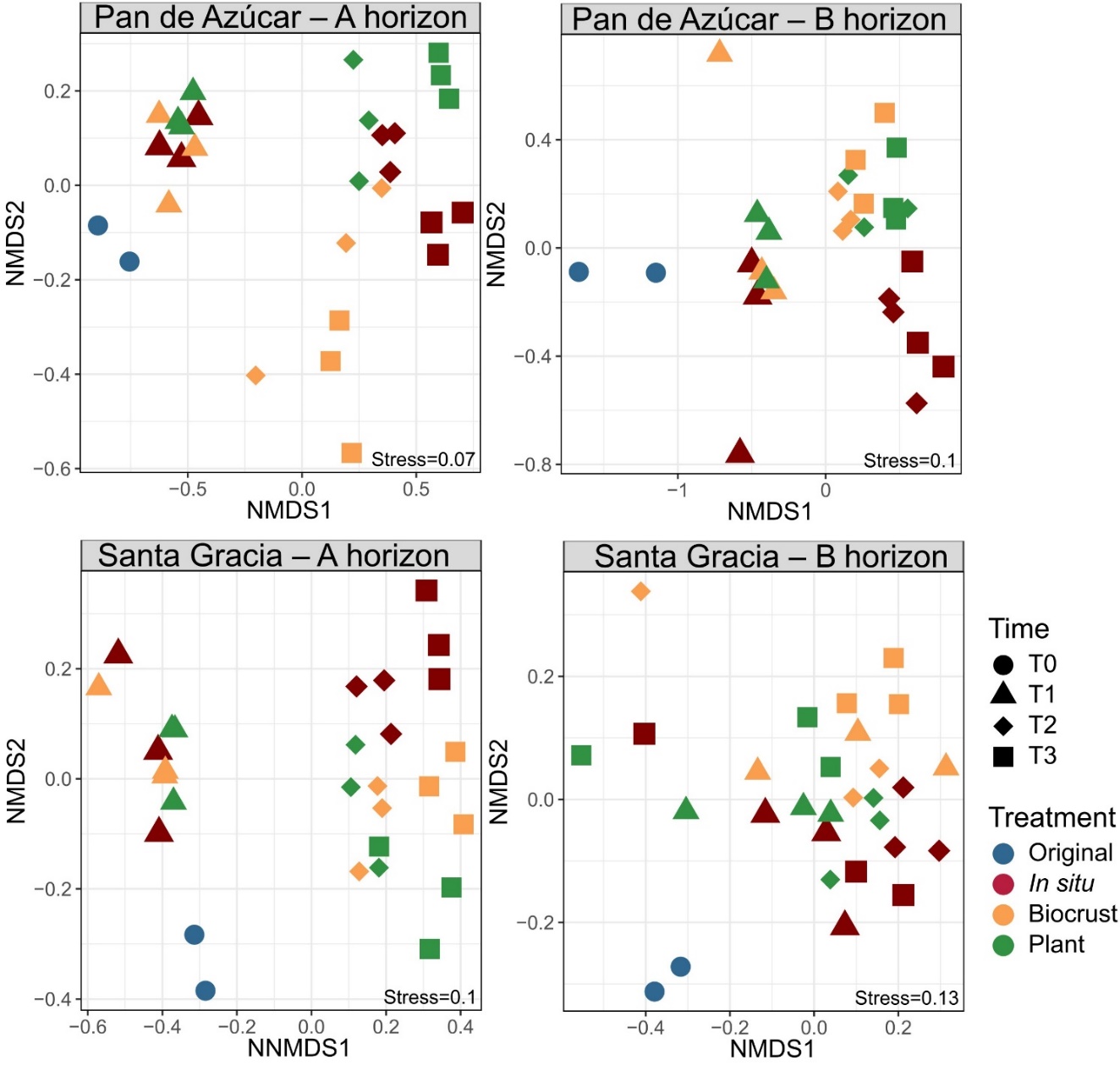


**Figure S4**. A nonmetric multidimensional scaling (NMDS) plot shows bacteria community shifts in Pan de Azúcar and Santa Gracia in four soil manipulation experiment time points. Time is represented by T0 (original), T1 (2 weeks), T2 (12 weeks), and T3 (16 weeks). Different color represents different treatments, while shapes represent different sampling times. Each point is the mean of biological triplicates.


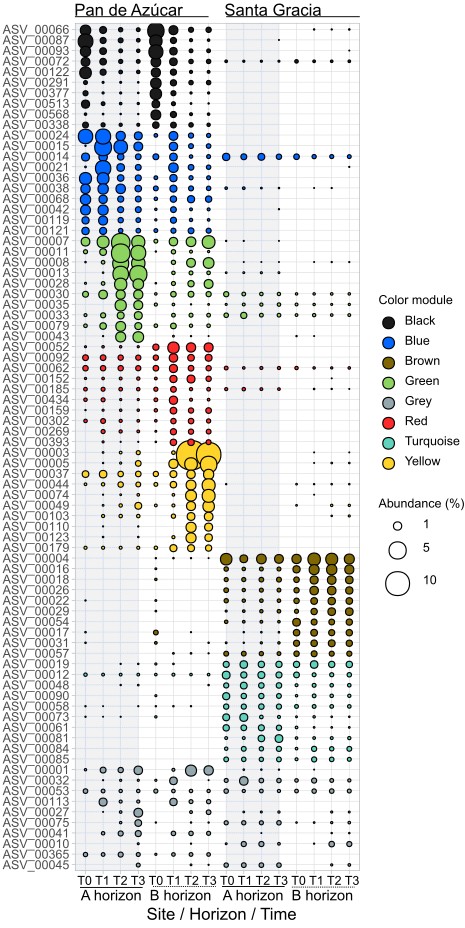


**Figure S5**. Top 70 relative abundance of ASV of the co-occurrence network for Pan de Azúcar and Santa Gracia in four-time points of the soil manipulation experiment. Colors represented different modules determined using the WGCNA method. Time is indicated as T0 (original), T1 (2 weeks), T2 (12 weeks), and T3 (16 weeks). Each bubble is the mean of the different treatments (*in situ*, BSCs, and plants) and biological triplicates.
